# Neural circuits expressing the serotonin 2C receptor regulate memory in mice and humans

**DOI:** 10.1126/sciadv.adl2675

**Published:** 2024-06-28

**Authors:** Hesong Liu, Yang He, Hailan Liu, Bas Brouwers, Na Yin, Katherine Lawler, Julia M. Keogh, Elana Henning, Dong-Kee Lee, Meng Yu, Longlong Tu, Nan Zhang, Kristine M. Conde, Junying Han, Zili Yan, Nikolas A. Scarcelli, Lan Liao, Jianming Xu, Qingchun Tong, Hui Zheng, Zheng Sun, Yongjie Yang, Chunmei Wang, Yanlin He, I. Sadaf Farooqi, Yong Xu

**Affiliations:** ^1^ USDA/ARS, Children’s Nutrition Research Center, Department of Pediatrics, Baylor College of Medicine, Houston, TX 77030, USA.; ^2^ Jan and Dan Duncan Neurological Research Institute, Texas Children’s Hospital, Houston, TX 77030, USA.; ^3^ Department of Pediatrics, Baylor College of Medicine, One Baylor Plaza, Houston, TX 77030, USA.; ^4^ University of Cambridge Metabolic Research Laboratories and NIHR Cambridge Biomedical Research Centre, Wellcome-MRC Institute of Metabolic Science, Addenbrooke's Hospital, Cambridge CB2 0QQ, UK.; ^5^ Department of Molecular and Cellular Biology, Baylor College of Medicine, Houston, TX 77030, USA.; ^6^ Brown Foundation Institute of Molecular Medicine, University of Texas Health Science Center at Houston, Houston, TX 77030, USA.; ^7^ Huffington Center on Aging, Baylor College of Medicine, Houston, TX 77030, USA.; ^8^ Department of Medicine, Baylor College of Medicine, Houston, TX 77030, USA.; ^9^ Pennington Biomedical Research Center, Brain Glycemic and Metabolism Control Department, Louisiana State University, Baton Rouge, LA 70808, USA.

## Abstract

Declined memory is a hallmark of Alzheimer’s disease (AD). Experiments in rodents and human postmortem studies suggest that serotonin (5-hydroxytryptamine, 5-HT) plays a role in memory, but the underlying mechanisms are unknown. Here, we investigate the role of 5-HT 2C receptor (5-HT_2C_R) in regulating memory. Transgenic mice expressing a humanized *HTR2C* mutation exhibit impaired plasticity of hippocampal ventral CA1 (vCA1) neurons and reduced memory. Further, 5-HT neurons project to and synapse onto vCA1 neurons. Disruption of 5-HT synthesis in vCA1-projecting neurons or deletion of 5-HT_2C_Rs in the vCA1 impairs neural plasticity and memory. We show that a selective 5-HT_2C_R agonist, lorcaserin, improves synaptic plasticity and memory in an AD mouse model. Cumulatively, we demonstrate that hippocampal 5-HT_2C_R signaling regulates memory, which may inform the use of 5-HT_2C_R agonists in the treatment of dementia.

## INTRODUCTION

Serotonin (5-hydroxytryptamine, 5-HT) is primarily synthesized by neurons in the dorsal Raphe nucleus (DRN) and median Raphe nucleus (MRN) in the midbrain, which project to multiple brain regions including the hippocampus, which is essential for short-term (working) and long-term memory formation. Restriction of dietary l-tryptophan (which is required for 5-HT synthesis) impairs short-term and long-term memory in rodents and humans (*1*, *2*). In addition, depletion of 5-HT during synaptogenesis (using a tryptophan hydroxylase inhibitor) decreases synaptic density in the adult rat hippocampus (*3*). Positron emission tomography studies have shown that people with mild cognitive impairment have reduced serotonin transporter availability (*4*, *5*), suggesting that loss of serotonin is associated with memory loss. However, the mechanisms by which serotonin regulates working memory are largely unknown.

Serotonin signals through multiple receptors, some of which have been implicated in memory (*6*–*11*), mood, behavior, and weight regulation (*12*–*14*). As serotonin 2C receptors (5-HT_2C_Rs) are abundantly expressed in the ventral hippocampal CA1 region (vCA1) (*15*) and have been implicated in cognition in rodents (*16*–*19*), we investigated the role of 5-HT_2C_Rs in memory in a series of studies in mice and humans.

## RESULTS

### Human 5-HT_2C_Rs mutations associated with impaired short-term memory

In a recent genetic study, we identified rare heterozygous loss-of-function (LOF) variants in *HTR2C*, which encodes 5-HT_2C_R in people with severe obesity (*20*). Here, five young women (22 to 29 years) carrying rare LOF *HTR2C* mutations completed the Prospective-Retrospective Memory Questionnaire (PRMQ) (*21*), and all reported remarkable impact on prospective and retrospective memory (table S1), with PRMQ T scores in the range associated with mild cognitive impairment (*22*); none had symptoms or a diagnosis of mood disorder at the time of testing. Variant carriers did not consent to more comprehensive cognitive testing, which would be needed to fully understand their phenotype.

We next asked whether functional variants in *HTR2C* affect aspects of memory in a large population-based cohort by studying 100,000 participants in the UK Biobank study with cognitive testing. We investigated carriers of 12 rare variants (allele frequency <0.1%) in *HTR2C* previously shown to cause a LOF in cells (*20*). We observed a reduced rate of prospective recall among males carrying T419A, which was among the most prevalent of the tested variants (table S2; “not recalled” versus “correct recall on [first or second] attempt”, odds ratio (95% confidence intervals) = 3.4 (1.4 to 7.3); 8 of 58 carriers (13.8%) and 2204 of 49,419 noncarriers (4.5%) did not recall; *P* = 0.0042, Fisher’s exact). These findings are preliminary and indicate the need for larger studies of people carrying *HTR2C* variants, which have been functionally characterized and on whom objective cognitive testing has been performed.

### Impaired memory in mice with a humanized *Htr2c* mutation

**Fig. 1.**
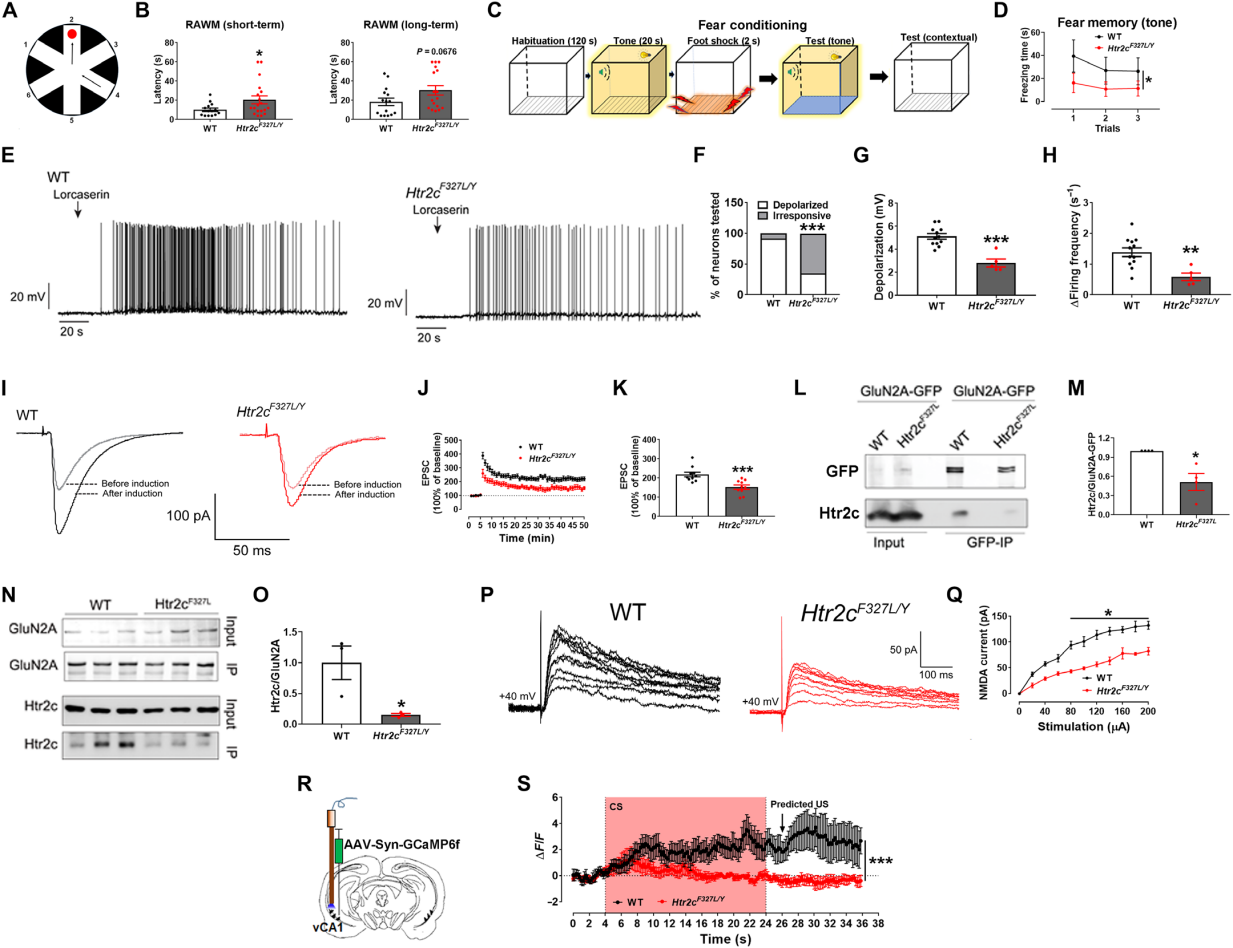
*Htr2c^F327L/Y^* mutation impairs memory and hippocampal neural plasticity in mice. (**A**) A schematic of RAWM apparatus. (**B**) Performance of WT and *Htr2c^F327L/Y^* mice in the RAWM test (*N* = 14 to 19 mice). (**C**) A schematic of fear conditioning training and test. (**D**) Performance of WT and *Htr2c^F327L/Y^* mice in the fear conditioning test (*N* = 16 or 21 mice). (**E**) Representative current clamp traces in vCA1 neurons treated with lorcaserin (100 μM, 5-s puff). (**F**) Percentage of neurons depolarized by lorcaserin (≥2 mV depolarization, *N* = 13 or 14 neurons from three mice per group). (**G** and **H**) Magnitude of lorcaserin-induced depolarization (G) and increase in firing frequency (H) only in depolarized neurons (*N* = 12 or 5 neurons from three mice per group). (**I**) Representative EPSC traces in vCA1 neurons. (**J**) Magnitude of EPSC elevations before and after LTP induction. (**K**) Averaged EPSC elevations during 45 to 50 min in (J) (*N* = 10 neurons from five mice per group). (**L**) Interaction of GluN2A-GFP and Htr2c protein in WT or F327L mutant cells. (**M**) Quantification of data in (L) (*N* = 4 biological replicates per group). (**N**) Interaction of GluN2A and Htr2c protein in the vCA1. (**O**) Quantification of data in (N) (*N* = 3 mice per group). (**P**) Representative NMDA currents in vCA1 neurons. (**Q**) Magnitude of NMDA currents (*N* = 12 or 5 neurons from three mice per group). (**R**) A schematic of fiber photometry recording of neuron activity of vCA1 neurons. (**S**) Temporal changes in vCA1 neuron activity in WT and *Htr2c^F327L/Y^* mice (6 months) in the tone-cued fear memory test (*N* = 13 or 14 mice). Results shown as means ± SEM. *P < 0.05, **P < 0.01, and ***P < 0.001 in two-sided unpaired *t* test [(B), (G), (H), (K), (M), and (O)], in two-way analysis of variance (ANOVA) [(D), (Q), and (S)], and in χ^2^ test (F).

The potential association between *HTR2C* variants and memory deficits observed in humans prompted us to use a humanized *Htr2c* mutant mouse model to further examine the causality. As we reported previously (*20*), among the LOF *HTR2C* variants we identified in humans, F327L renders the most severe impairment to the 5-HT_2C_R signaling and we have used the CRISPR-Cas9 approach to make a knock-in *Htr2c^F327L^* mouse line. Here, we confirmed that *Htr2c^F327L^* mice do not have off-target mutations (fig. S1), and we next examined whether these mice have impaired memory. In the radial arm water maze (RAWM), we found that compared to wild-type (WT) mice, male, chow-fed, and weight-matched *Htr2c^F327L/Y^* mice showed comparable learning curves during the 12 training sessions (Fig. 1A and fig. S2, A and B). During the short-term memory test that took place 30 min after the last training session, male *Htr2c^F327L/Y^* mice showed significantly increased latency in finding the platform (Fig. 1B). We also tested long-term memory in the same apparatus after 24 hours and detected a trend toward increased latency in male *Htr2c^F327L/Y^* mice (Fig. 1B). There was no difference in swimming speed in the water maze between the two groups (fig. S2C). In a fear conditioning test, male *Htr2c^F327L/Y^* mice exhibited significantly reduced freezing behavior in response to tone cues conditioned with foot shock stimuli (Fig. 1, C and D), but their responses to the contextual cues were comparable to WT male mice (fig. S2D). We further confirmed that male *Htr2c^F327L/Y^* mice had a similar pain threshold to male WT mice in the hot plate test (fig. S2E).

We also examined a cohort of female chow-fed and weight-matched WT and *Htr2c^F327L/+^* mice (fig. S2F). Compared to female WT mice, female *Htr2c^F327L/+^* mice showed significantly impaired learning during RAWM training and impaired short-term memory but comparable long-term memory and swimming speed (fig. S2, G to I). In the fear conditioning test, female *Htr2c^F327L/+^* mice showed comparable freezing behavior in response to either tone or contextual cues (fig. S2, J and K). We then examined effects of systemic administration of a 5-HT_2C_R antagonist (SB242084, 1 mg kg^−1^, 14 days) (*23*) in a cohort of male chow-fed WT mice. SB242084 significantly impaired short-term and long-term memory in the RAWM test without changing body weight, swimming speed, or performance in the fear conditioning test (fig. S2, L to Q). In a female chow-fed WT mice, SB242084 significantly impaired long-term memory in the RAWM test without changing other parameters (fig. S2, R to W).

We next examined the neural basis for the observed impairment in memory. We found that the 5-HT_2C_R agonist, lorcaserin (*24*), activated most vCA1 neurons from male WT mice, causing depolarization and increased firing frequency, but this effect was blunted in male *Htr2c^F327L/Y^* mice (Fig. 1, E to H, and fig. S3A). Notably, lorcaserin did not activate neurons in the dorsal CA1 (dCA1, fig. S3B). Activity-dependent long-lasting changes in synaptic efficacy in CA3-CA1 synapse in the hippocampus, such as long-term potentiation (LTP), are essential for memory formation. vCA1 pyramidal neurons receive extensive projection from CA3 region (*25*, *26*). Thus, we then recorded evoked excitatory postsynaptic currents (EPSCs) in vCA1 pyramidal neurons at the synapses substantially involving CA3-vCA1 projections using a high-frequency field stimulation protocol (*27*, *28*) to induce LTP of EPSC in vCA1 neurons. We found that LTP in the vCA1 was blunted in male *Htr2c^F327L/Y^* mice compared to WT mice (Fig. 1, I to K, and fig. S3, C to H). Notably, the LTP detected in the dorsal CA1 neurons was comparable between WT and *Htr2c^F327L/Y^* mice (fig. S3, I to K). These findings indicate that 5-HT_2C_R signaling regulates LTP, a prerequisite of synaptic plasticity, in vCA1 hippocampal neurons.

5-HT_2C_Rs are known to form a protein complex with the *N*-methyl-D-aspartate (NMDA) glutamate receptor subunit, GluN2A, and facilitate NMDA activation (*29*). We consistently detected the protein-protein interaction between WT 5-HT_2C_R and GluN2A in human embryonic kidney (HEK) 293T cells and found that this interaction was significantly attenuated by the F327L mutation (Fig. 1, L and M). We further confirmed that the 5-HT_2C_R protein interacted with GluN2A protein in the vCA1 of male WT mice, which was significantly attenuated in the vCA1 of male *Htr2c^F327L/Y^* mice (Fig. 1, N and O). NMDA currents, which play a critical role in the formation of EPSC LTP (*30*), were also significantly attenuated in vCA1 neurons from *Htr2c^F327L/Y^* mice (Fig. 1, P and Q), whereas AMPA currents were not affected (fig. S3, L and M). NMDA receptor (NMDAR)–independent LTP, recorded in the presence of D-AP5 (an NMDAR inhibitor), was comparable between vCA1 neurons from the two groups (fig. S3, N to P). Thus, these results support that 5-HT_2C_R signals facilitate NMDA currents in vCA1 neurons, which likely underlies the development of EPSC LTP.

To further examine the relevance of vCA1 neurons in memory regulation, we stereotaxically injected adeno-associated virus (AAV)–Syn-GCaMP6f into the vCA1 of male WT or *Htr2c^F327L/Y^* mice (Fig. 1R) and implanted an optic fiber to allow fiber photometry recording of vCA1 neuron activity in freely moving mice. These mice were then subjected to fear conditioning training sessions, followed by a tone memory test, as described above (Fig. 1C). During the test, the tone [conditioned stimulus (CS)] induced increases in vCA1 neuron activities in both WT and *Htr2c^F327L/Y^* mice (Fig. 1S). vCA1 neurons from WT mice also exhibited increased activities at the time point when the foot shocks [unconditioned stimulus (US)] would have been given if it were a training session (Fig. 1S), suggesting that increased vCA1 neuron activity encodes the predicted US. However, vCA1 neurons in *Htr2c^F327L/Y^* mice did not show such increased activities (Fig. 1S), indicating that the 5-HT_2C_R signaling is required for vCA1 neurons to encode predicted US.

### Impaired memory in mice lacking 5-HT_2C_R in the vCA1

**Fig. 2.**
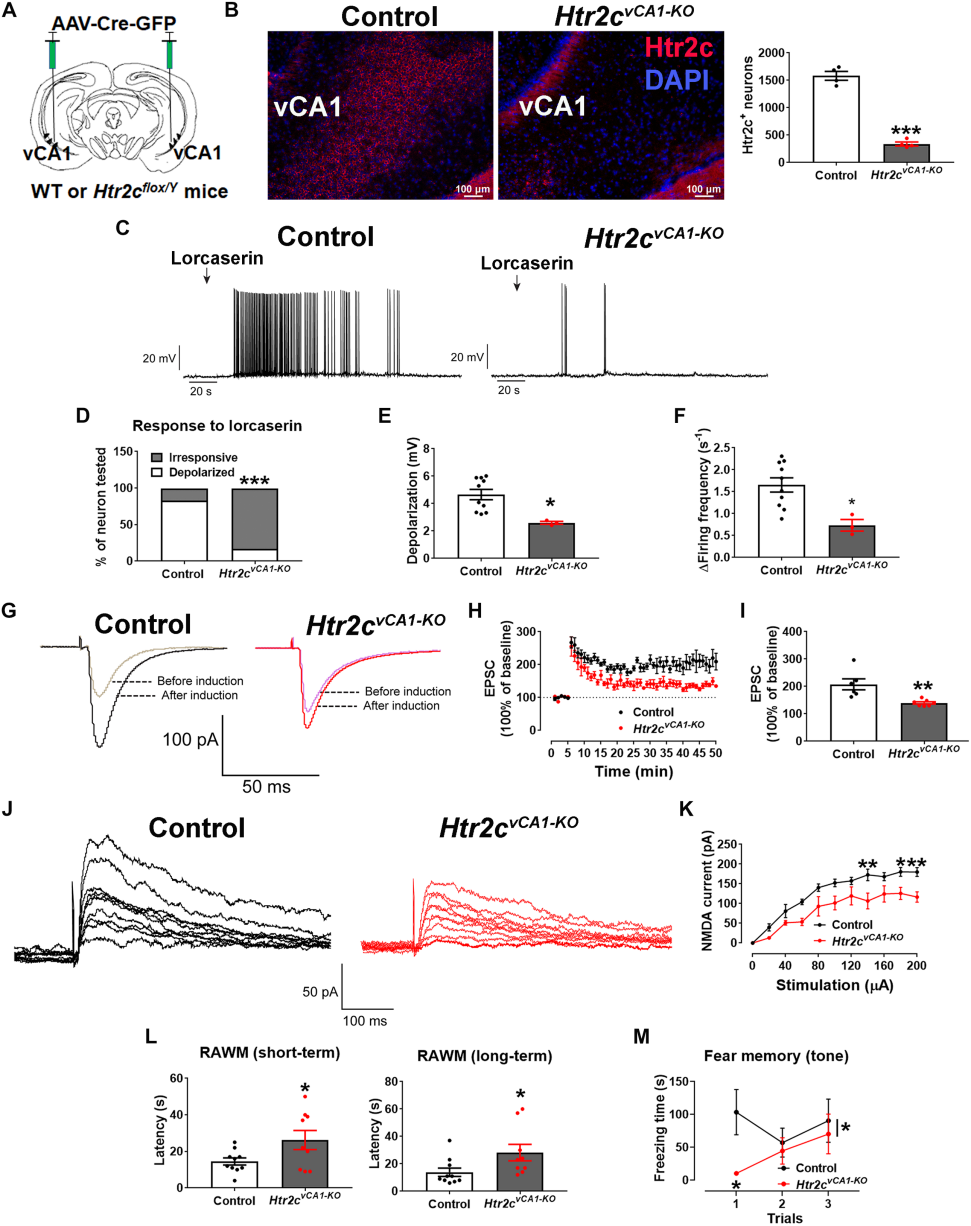
Inhibition of 5-HT_2C_Rs impairs vCA1 neural plasticity and cognition. (**A**) Generation of mice lacking 5-HT_2C_Rs in the vCA1 region of the hippocampus (*Htr2c^vCA1-KO^*) by injecting AAV-Cre-GFP into the vCA1 of Htr2c^flox/Y^ bilaterally. (**B**) Left, representative microscopic images showing the nuclear stain 4′,6-diamidino-2-phenylindole (DAPI, blue) and RNAscope signals for Htr2c (red) in the vCA1 of control or *Htr2c^vCA1-KO^* mice; scale bar, 100 μm. Right, quantification of Htr2c mRNAs in the vCA1 (*N* = 3 mice per group). (**C**) Representative current clamp traces in vCA1 neurons from control or *Htr2c^vCA1-KO^* mice when treated with lorcaserin (100 μM, 5-s puff). (**D**) Percentage of neurons that were depolarized by lorcaserin (≥2-mV depolarization, *N* = 12 or 17 neurons from three mice per group). (**E** and **F**) Magnitude of lorcaserin-induced depolarization (E) and increase in firing frequency (F) in depolarized neurons (*N* = 10 or 3 neurons from three mice per group). (**G**) Representative EPSC traces before and after LTP induction in vCA1 neurons from control or *Htr2c^vCA1-KO^* mice. (**H**) Magnitude of EPSC elevations before and after LTP induction. (**I**) Averaged EPSC elevations during 45 to 50 min in (H) (*N* = 6 or 7 neurons from three mice per group). (**J**) Representative NMDA current traces in vCA1 neurons from control or *Htr2c^vCA1-KO^* mice. (**K**) Magnitude of NMDA currents at different stimulations (*N* = 6 to 7 neurons from three mice per group). (**L**) Latency of control and *Htr2c^vCA1-KO^* mice to reach the platform in the RAWM test (*N* = 9 to 10 mice). (**M**) Freezing time of control and *Htr2c^vCA1-KO^* mice during the fear memory test in response to tone cues. Results are shown as means ± SEM. *P < 0.05, **P < 0.01, and ***P < 0.001 in two-sided unpaired *t* test [(B), (E), (F), (K), (I), and (L)], in two-way ANOVA [(K) and (M)], and in χ^2^ test (D).

To further test whether 5-HT_2C_R signaling is required for vCA1 neural plasticity, learning, and memory, we stereotaxically injected AAV-Cre–green fluorescent protein (GFP) into the vCA1 of male *Htr2c^flox/Y^* mice (*31*) to delete *Htr2c* in vCA1 neurons (Fig. 2, A and B). This deletion significantly blunted lorcaserin-induced depolarization and reduced LTP and NMDA currents in vCA1 neurons (Fig. 2, C to K). These mice exhibited deficits in learning, short-term and long-term memory in the RAWM test, impaired fear memory to the tone cues but normal fear memory to the contextual cues (Fig. 2, L and M, and fig. S4, A to D). In the open field test, male mice lacking 5-HT_2C_Rs in vCA1 neurons did not show significant changes in the center entry or time spent in the center or in the margin (fig. S4, E to H). Thus, these data demonstrate that the 5-HT_2C_R signaling in hippocampal vCA1 neurons is required for normal learning and memory.

### Midbrain 5-HT to the vCA1 projections

**Fig. 3.**
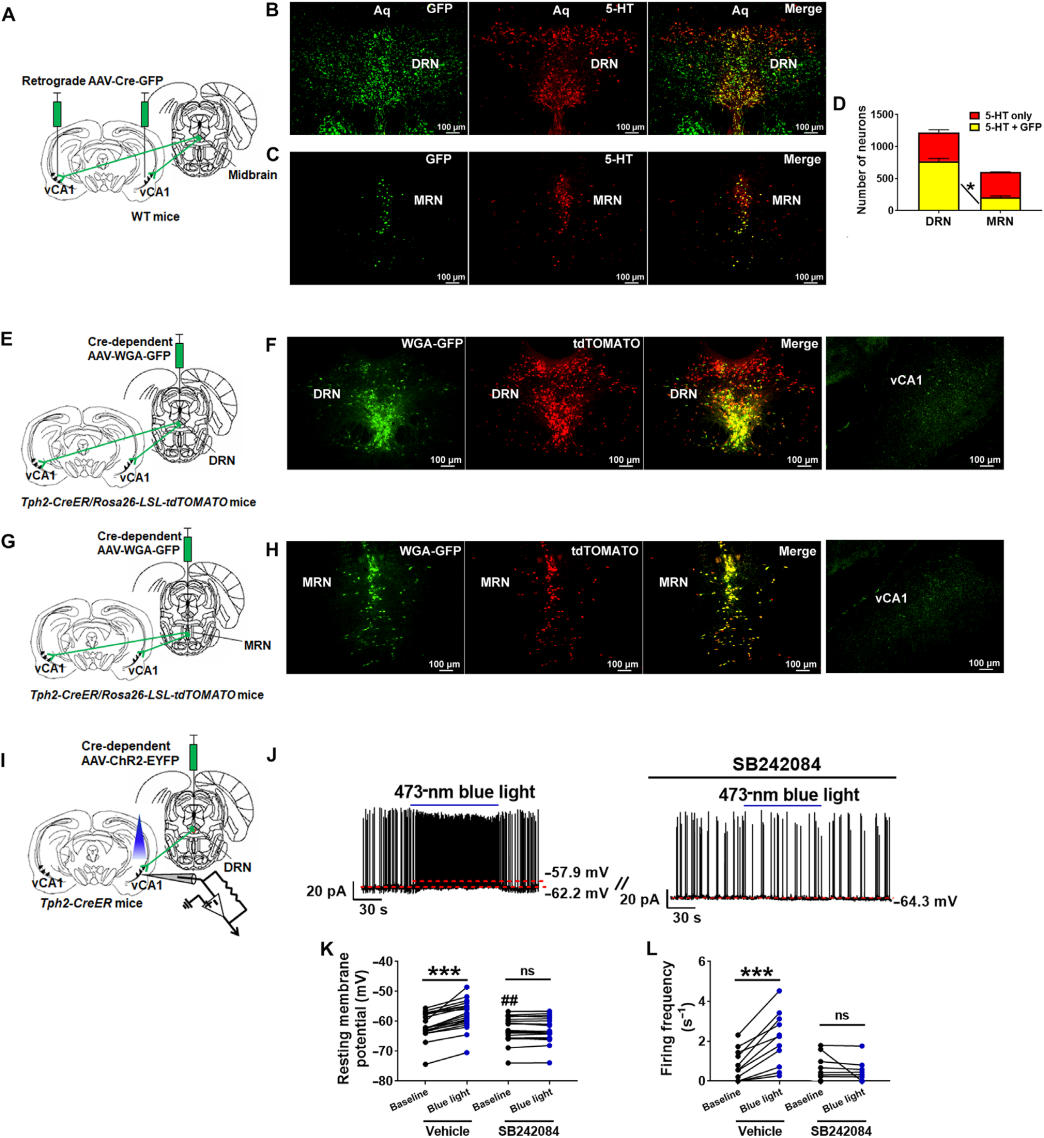
Midbrain 5-HT neurons project to and activate vCA1 neurons. (**A**) A schematic illustration of retrograde tracing from vCA1 neurons to midbrain 5-HT neurons using a retrograde AAV-Cre-GFP virus. (**B** and **C**) Representative microscopic images showing GFP (left), 5-HT (middle), and merge (right) in the DRN (B) and MRN (C); scale bars, 100 μm. (**D**) Quantification of neurons that were 5-HT positive only or were labeled by both 5-HT and GFP in the DRN and MRN. Results shown as means ± SEM. *P < 0.05 in double-labeled neurons in the DRN versus MRN in two-way ANOVA followed by Tukey’s test (*N* = 3 mice). (**E**) A schematic illustration of anterograde retrograde tracing from 5-HT^DRN^ neurons to vCA1 neurons using Cre-dependent AAV-WGA-GFP virus in *Tph2-CreER/Rosa26-LSL-tdTOMATO* mice. (**F**) Representative microscopic images showing WGA-GFP (left), tdTOMATO (left-middle), and merge (right-middle) in the DRN and WGA-GFP in the vCA1 (right); scale bars, 100 μm. (**G**) A schematic illustration of anterograde retrograde tracing from 5-HT^MRN^ neurons to vCA1 neurons using Cre-dependent AAV-WGA-GFP virus in *Tph2-CreER/Rosa26-LSL-tdTOMATO* mice. (**H**) Representative microscopic images showing WGA-GFP (left), tdTOMATO (left-middle), and merge (right-middle) in the MRN and WGA-GFP in the vCA1 (right); scale bars, 100 μm. (**I**) A schematic illustration of using CRACM to examine the 5-HT^DRN^ → vCA1 circuit. (**J**) Representative current clamp traces in vCA1 neurons in response to blue light pulses (473 nM, 40 mW, 10-ms pulse, 10 Hz for 2 min) in the absence or the presence of 100 μM of the selective 5-HT_2C_R receptor antagonist, SB242084. (**K** and **L**) RM potential (K) and firing frequency (L); ***P < 0.001 between baseline and blue light; ##*P* < 0.01 at the baseline between vehicle and SB242084 treated neurons (*N* = 11 to 20 neurons from three mice per group). ns, not significant.

The observations that loss of 5-HT_2C_Rs in the vCA1 resulted in impaired memory suggest that the vCA1 receives 5-HTergic inputs. To test this possibility, we stereotaxically injected a retrograde AAV-Cre-GFP virus into the vCA1 of male WT mice (Fig. 3A and fig. S5A). Ten days later, we detected abundant GFP-labeled neurons in the DRN and a modest number of GFP-labeled neurons in the MRN of the midbrain (Fig. 3, B to D). Costaining with 5-HT revealed that 63% 5-HT^DRN^ neurons and 34% 5-HT^MRN^ neurons were retrogradely labeled (Fig. 3, B to D), indicating that these midbrain 5-HT neurons project to the vCA1. Notably, 5-HT neurons in the caudal Raphe nuclei (CRN) in the hindbrain were not labeled by GFP (fig. S5B). To further examine the connectivity between midbrain 5-HT neurons and vCA1 neurons, we used a tryptophan hydroxylase 2 (*Tph2*)*–CreER* mouse line in which 5-HT neurons selectively express tamoxifen-inducible Cre recombinase (see fig. S5, C and D for validation) (*32*). We stereotaxically injected AAVDJ-DIO-WGA-GFP virus into the DRN of male *Tph2-CreER/Rosa26-LSL-tdTOMATO* mice (with tamoxifen induction) to selectively express WGA-GFP in 5-HT^DRN^ neurons (Fig. 3E). WGA-GFP anterogradely traveled along the 5-HT fibers, passed the synapse, and filled the downstream neurons that were innervated by 5-HT terminals (*33*, *34*). Four weeks after the infection, we perfused the mice and detected GFP-labeled cell bodies in the DRN (the injection site) and in the vCA1 (Fig. 3F). In a similar experiment where the AAVDJ-DIO-WGA-GFP virus was stereotaxically injected into the MRN of male *Tph2-CreER/Rosa26-LSL-tdTOMATO* mice, we observed GFP-labeled cell bodies in the MRN (the injection site) and in the vCA1 (Fig. 3, G and H). Thus, these results indicate that both 5-HT^DRN^ neurons and 5-HT^MRN^ neurons project to vCA1 neurons.

To test whether the circuit between 5-HT^DRN^ neurons and vCA1 neurons is monosynaptic, we performed channel rhodopsin–assisted circuit mapping (CRACM). We stereotaxically injected a Cre-dependent AAV vector carrying ChR2-EYFP into the DRN of male *Tph2-CreER* mice, which expressed ChR2-EYFP in 5-HT^DRN^ neurons (Fig. 3I). Photostimulation rapidly increased action potential firing frequency and resting membrane (RM) potential in 91% tested vCA1 neurons; these responses were markedly reduced by the selective 5-HT_2C_R antagonist, SB242084 (Fig. 3, J to L). Cumulatively, these results demonstrate that 5-HT^DRN^ neurons provide 5-HTergic inputs to activate most vCA1 neurons via 5-HT_2C_R–mediated mechanisms.

Since 5-HT^DRN^ neurons may corelease glutamate and/or γ-aminobutyric acid (GABA) (*35*, *36*), we examined postsynaptic currents induced by these neurotransmitters. We detected blue light–evoked EPSCs in 63% of tested vCA1 neurons (fig. S6A), which were blocked by glutamate receptor inhibitors (fig. S6, B to D). We also detected light-evoked inhibitory postsynaptic currents (IPSCs) in 25% tested vCA1 neurons (fig. S6E), which were blocked by a GABA type A (GABA_A_) receptor inhibitor (fig. S6, F to H). These results indicate that in addition to 5-HT, 5-HT^DRN^ neurons provide glutamatergic and/or GABAergic inputs to a subset of vCA1 neurons.

### 5-HT➔vCA1 projections and memory

**Fig. 4.**
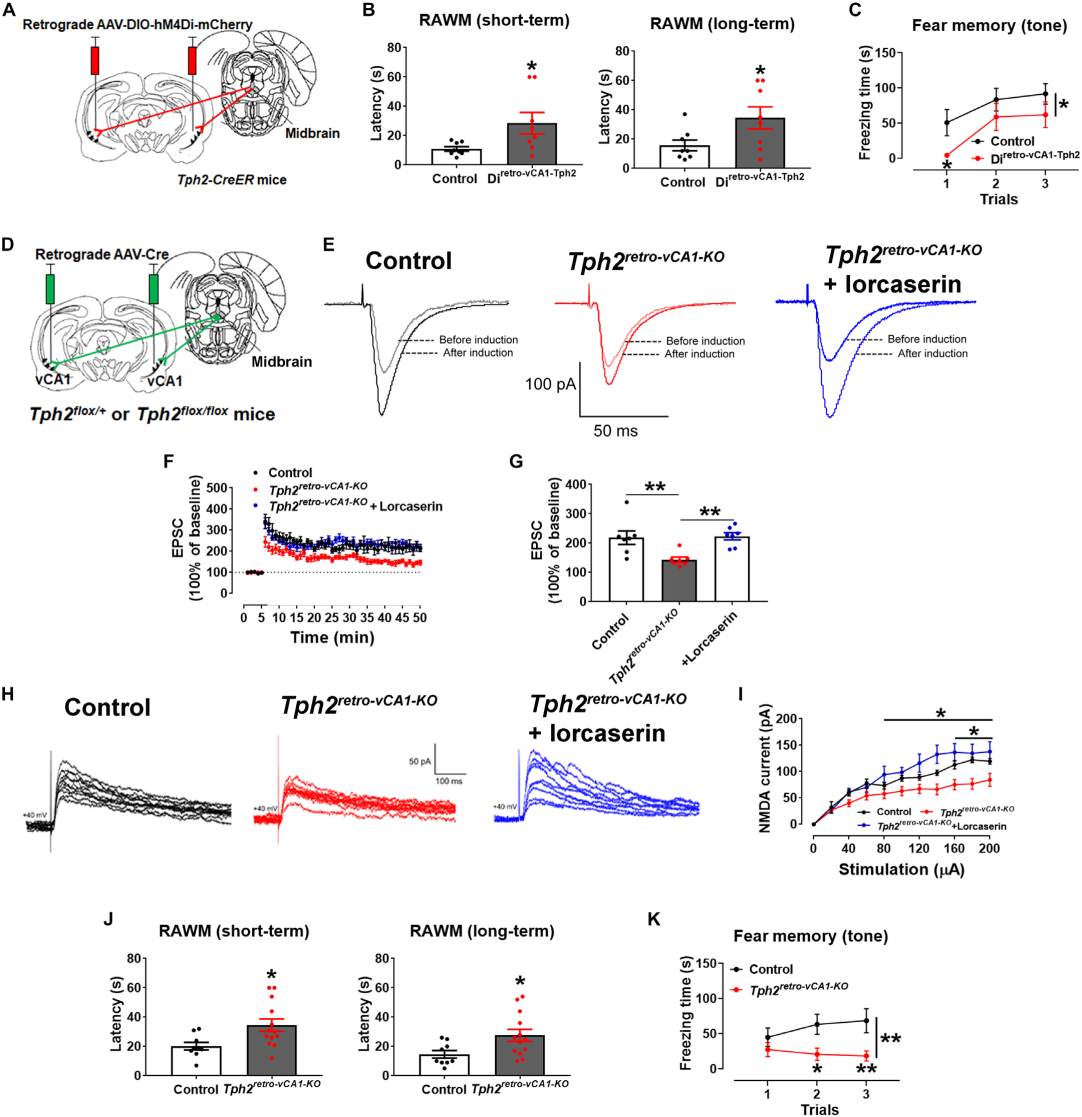
5-HTergic projections to the vCA1 regulate neural plasticity and cognitive function. (**A**) Generation of mice to chemogenetically inhibit 5-HTergic projections to the vCA1. (**B**) Latency of CNO-treated control and *Di^retro-vCA1-Tph2^* mice to reach the platform in the RAWM test (*N* = 8 mice). (**C**) Freezing time of weight-matched control and *Htr2c^vCA1-KO^* mice during the fear memory test in response to tone cues (*N* = 8 mice). (**D**) Generation of mice lacking 5-HT synthesis only in vCA1-projecting 5-HT neurons and their controls. (**E**) Representative EPSC traces before and after LTP induction in vCA1 neurons from control and *Tph2^retro-vCA1-KO^* mice with or without lorcaserin treatment (10 μM). (**F**) Magnitude of EPSC elevations before and after LTP induction. (**G**) Averaged EPSC elevations during 45 to 50 min in (F) (*N* = 7 neurons from three mice per group). (**H**) Representative NMDA currents in vCA1 neurons from control and *Tph2^retro-vCA1-KO^* mice with or without lorcaserin treatment (10 μM). (**I**) Magnitude of NMDA currents (*N* = 7 or 8 neurons from three mice per group). (**J**) Latency of weight-matched control and *Tph2^retro-vCA1-KO^* mice to reach the platform in the RAWM test (*N* = 9 or 13 mice). (**K**) Freezing time of weight-matched control and *Tph2^retro-vCA1-KO^* mice during the fear memory test in response to tone cues (*N* = 10 or 14 mice). Results are shown as means ± SEM. *P < 0.05 and **P < 0.01 in two-sided unpaired *t* test [(B) and (J)], in one-way ANOVA (G), and in two-way ANOVA [(C), (I), and (K)].

To further examine the function of the 5-HT➔vCA1 circuit on memory, we injected a retrograde Cre-dependent AAV-hM4Di virus into the vCA1 of male *Tph2-CreER* mice to allow clozapine N-oxide (CNO)-mediated chemogenetic inhibition of 5-HT neurons that project to the vCA1 (Di^retro-vCA1-Tph2^, Fig. 4A). Compared to CNO-treated controls (WT mice receiving the same virus injections), CNO-treated Di^retro-vCA1-Tph2^ mice showed significantly impaired short-term and long-term memory in the RAWM test and impaired fear memory to the tone cues but not to contextual cues (Fig. 4, B and C, and fig. S7, A to D).

To confirm whether the memory function of the 5-HT➔vCA1 circuit depends on 5-HT, we injected a retrograde AAV vector carrying Cre into the vCA1 of male *Tph2^flox/flox^* mice, in which the 5-HT–synthesizing enzyme Tph2 can be selectively deleted in a Cre-dependent manner. Upon uptake by the terminals in the vCA1, this AAV vector traveled retrogradely to delete floxed alleles in neurons that directly project to the vCA1 (*Tph2^retro-vCA1-KO^*, Fig. 4D and fig. S7, E to H). *Tph2^retro-vCA1-KO^* mice lacking 5-HT synthesis in vCA1-projecting 5-HT neurons showed significantly blunted LTP and NMDA currents in vCA1 neurons (Fig. 4, E to I). Lorcaserin, when added to the brain slices from *Tph2^retro-vCA1-KO^* mice, was able to rescue both LTP and NMDA currents (Fig. 4, E to I). In addition, these *Tph2^retro-vCA1-KO^* mice also showed significantly impaired short-term and long-term memory in the RAWM test and impaired fear memory to the tone cues but not to contextual cues (Fig. 4, J and K, and fig. S7, I to L). Together, these results indicate that activation of vCA1-projecting 5-HT neurons is required to maintain normal memory function, likely through 5-HT release into the vCA1.

### Effects of 5-HT_2C_R agonism in a mouse model of amyloid pathology

**Fig. 5.**
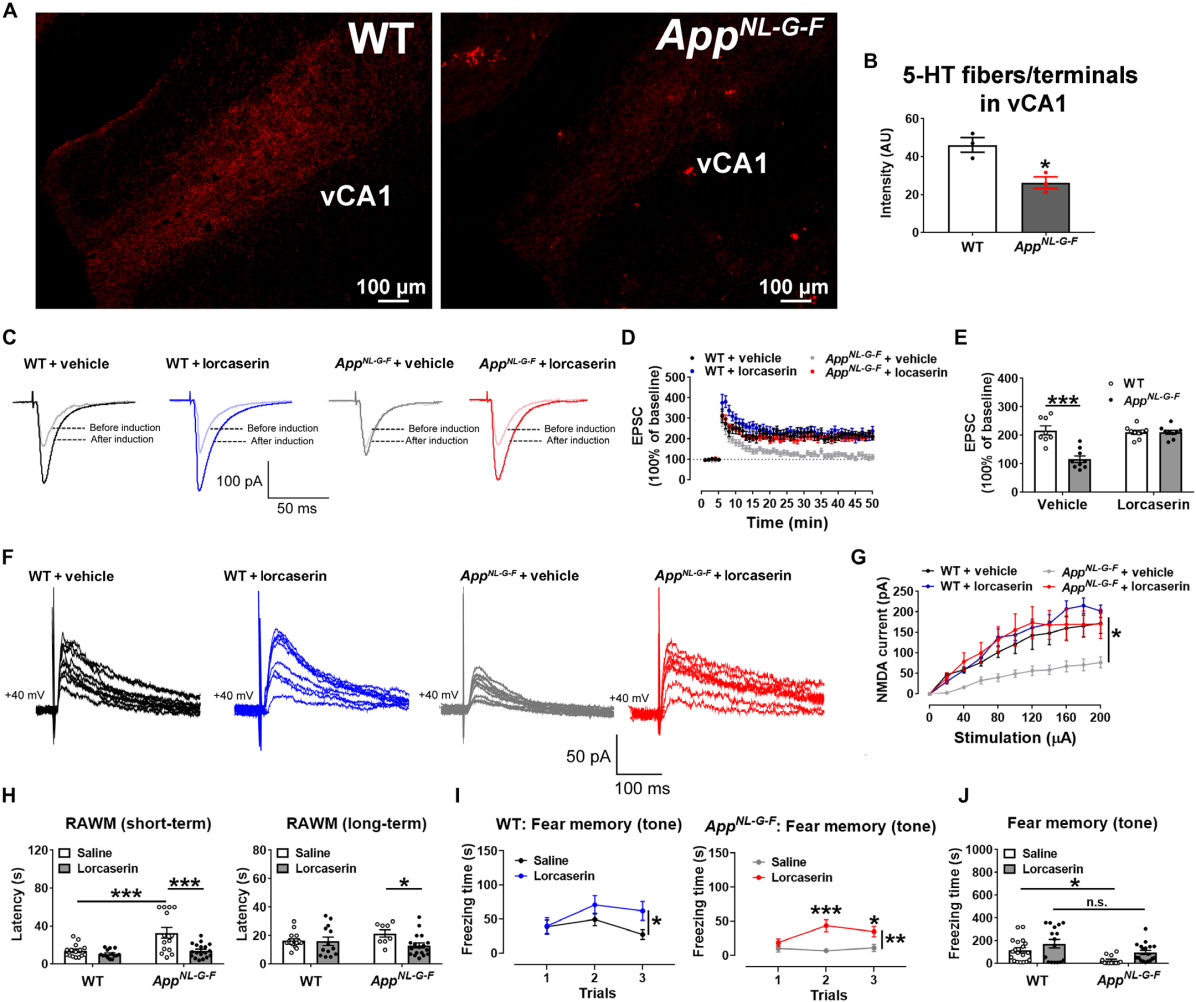
The 5-HT_2C_R agonist improves vCA1 neural plasticity and memory in *App^NL-G-F^* mice. (**A**) Representative microscopic images showing 5-HTergic fibers/terminals (labeled through Tph2-CreER/Rosa26-LSL-tdTOMATO allele induced by tamoxifen at 8 weeks) in the vCA1 of 6-month-old WT or *App^NL-G-F^* mice. (**B**) tdTOMATO intensity in the vCA1 (*N* = 3 mice per group). (**C**) Representative EPSC traces before and after LTP induction in vCA1 neurons from WT and *App^NL-G-F^* mice incubated with vehicle or lorcaserin. (**D**) Magnitude of EPSC elevations before and after LTP induction. (**E**) Averaged EPSC elevations during 45 to 50min in (D) (*N* = 8 or 9 neurons from five mice per group). (**F**) Representative NMDA current traces in vCA1 neurons from WT and *App^NL-G-F^* mice incubated with vehicle or lorcaserin. (**G**) Magnitude of NMDA currents at different stimulations. Results shown as means ± SEM. The “WT + Vehicle” group was significantly different from each of three other groups (*N* = 8, 9, or 10 neurons from three mice per group). (**H**) Latency of saline or lorcaserin-treated WT and *App^NL-G-F^* mice (6 months) to reach the platform in the RAWM test (*N* = 14, 15, or 18 mice). (**I**) Freezing time of saline- or lorcaserin-treated WT and *App^NL-G-F^* mice (6 months) during the fear memory test in response to tone cues (*N* = 12, 16, 18, or 19 mice). (**J**) Sum of freezing time in (I) in each group (*N* = 12, 16, 18, or 19 mice). Results are shown as means ± SEM. *P < 0.05, **P < 0.01, and ***P < 0.001 in two-sided unpaired *t* test (B) and in two-way ANOVA [(E), (G), (H), (I), and (J)]. AU, arbitrary units.

Given the important role of the 5-HT➔vCA1 circuit in memory regulation, we then sought to examine the anatomical integrity of the 5-HT➔vCA1 circuit in a mouse model with known memory deficits due to humanized mutations in the amyloid precursor protein (APP), *App^NL-G-F^* knock-in mice (*37*). To this end, *Tph2-CreER/Rosa26-LSL-tdTOMATO* alleles were crossed onto WT or *App^NL-G-F^* mice, and we induced *Tph2-CreER* activity with tamoxifen at 8 weeks of age to label 5-HT neurons with tdTOMATO. At 6 months of age, when *App^NL-G-F^* mice are known to display memory impairment (*37*), we detected a reduction of tdTOMATO-labeled cell bodies in the DRN of male *App^NL-G-F^* mice compared to male WT mice, whereas tdTOMATO-labeled cell bodies in the MRN were comparable (fig. S8, A to C). We also observed a significant reduction of tdTOMATO-labeled fibers/terminals in the vCA1 of *App^NL-G-F^* mice compared to WT mice (Fig. 5, A and B). These data indicate that amyloid pathology can damage 5-HT➔vCA1 projections.

Consistent with the damaged 5-HT innervations in the vCA1, vCA1 neurons from vehicle-incubated male *App^NL-G-F^* brain slices (6 months of age) showed significantly blunted LTP and NMDA currents compared to those from vehicle-incubated male WT slices, which were restored by lorcaserin incubation (Fig. 5, C to G). Further, we showed that lorcaserin-induced excitations were comparable in vCA1 neurons from male WT versus *App^NL-G-F^* slices (fig. S8, D to F). On the other hand, the 5-HT_2C_R antagonist, SB242084, significantly reduced the LTP in WT vCA1 neurons but did not further reduce LTP in male *App^NL-G-F^* vCA1 neurons (fig. S8, G to I). Together, these data indicate that impaired neural plasticity in vCA1 neurons caused by amyloid pathology can be restored by enhanced 5-HT_2C_R signals.

We then examined whether pharmacological activation of 5-HT_2C_Rs can improve memory in *App^NL-G-F^* mice. Male WT and *App^NL-G-F^* littermates (at the age of 5 months and 3 weeks) were primed with daily injections of saline or lorcaserin for 2 weeks. Then, while they continued to receive daily injections, they were subjected to the RAWM and fear conditioning tests. Lorcaserin administration in male *App^NL-G-F^* mice significantly improved short-term and long-term memory in the RAWM test and improved fear memory to the tone cues but not to contextual cues (Fig. 5, H to J, and fig. S8, J to L). Notably, we chose to use a low dose of lorcaserin (1 mg per kg body weight per day), which did not significantly alter body weight, swim speed, and sleep (fig. S8, M to O). Thus, these results indicate that the selective 5-HT_2C_R agonist can improve memory in 6-month-old *App^NL-G-F^* mice, an effect that is independent of effects on body weight (*24*, *38*). In addition, EPSC LTP in vCA1 neurons was significantly blunted in saline-injected male *App^NL-G-F^* mice compared to saline-injected male WT mice, which was restored in lorcaserin-injected male *App^NL-G-F^* mice (fig. S8, P to R).

We also tested whether chemogenetic activation of the 5-HT➔vCA1 circuit can improve memory in 6-month-old *App^NL-G-F^* mice. To this end, the *Tph2-CreER* allele was crossed onto *App^NL-G-F^* mice, and we then stereotaxically injected a retrograde Cre-dependent AAV-hM3Dq virus into the vCA1 of these male *App^NL-G-F^/Tph2-CreER* mice to allow CNO-mediated chemogenetic activation of 5-HT neurons that project to the vCA1. Compared to CNO-treated male *App^NL-G-F^* mice (without *Tph2-CreER* but receiving the same virus injections), CNO-treated Dq^retro-vCA1-Tph2^ mice showed no changes in performances in the RAWM and fear conditioning tests (fig. S8, S to X), indicating that activation of the damaged 5-HT➔vCA1 projections in 6-month-old *App^NL-G-F^* mice is not sufficient to improve the cognitive function, likely due to the substantial reduction of 5-HTergic fibers/terminals in the vCA1 of 6-month-old *App^NL-G-F^* mice (Fig. 5, A and B).

## DISCUSSION

Here, we report that human LOF *HTR2C* mutations are associated with learning and memory deficits. Our studies suggest that cognitive phenotypes associated with impaired 5-HT_2C_R signaling may be more readily identified in male (hemizygous, one X chromosome) rather than female variant carriers (heterozygous, two X chromosomes). Further studies involving the functional characterization of all *HTR2C* variants found in large cohorts with comprehensive cognitive testing are needed.

We show that mice carrying a severe LOF human *HTR2C* mutation have impaired memory and impaired plasticity of hippocampal vCA1 neurons. We delineated a 5-HT➔vCA1 circuit and found that 5-HT_2C_Rs mediate neurotransmission through this circuit. Using mice with the 5-HT➔vCA1 circuit chemogenetically inhibited, mice lacking 5-HT synthesis in vCA1-projecting 5-HT neurons and mice lacking 5-HT_2C_Rs in the vCA1, we demonstrated the physiological significance of 5-HT/5-HT_2C_R signals in vCA1 neural plasticity and memory formation. By demonstrating that a selective 5-HT_2C_R agonist, lorcaserin, can improve the impaired vCA1 neural plasticity and memory loss in *App^NL-G-F^* mice, we identified 5-HT_2C_Rs as a rational target for therapeutic intervention in dementia. Notably, lorcaserin was originally developed to reduce body weight (*24*) and was approved by the Food and Drug Administration (FDA) in 2012 as an anti-obesity medicine [although withdrawn in 2020 because of a potential cancer risk (*39*)]. Our results provide proof of concept to support the use of 5-HT_2C_R agonism as a rational strategy to improve memory loss in patients with amyloid pathology. Of note, emerging evidence indicates that 5-HT dysregulation is also associated with tau phosphorylation (*40*, *41*). Whether lorcaserin can also improve memory in mouse models of tau pathology warrants further investigation.

Our results support a model in which 5-HT released from midbrain 5-HT neurons acts upon 5-HT_2C_Rs expressed by vCA1 neurons to trigger EPSC LTP, potentially by enhancing responsiveness to glutamatergic inputs. 5-HT_2C_Rs are known to form a protein complex with the NMDA glutamate receptor and facilitate NMDA activation (*29*). Here, we reproduced the protein-protein interaction between the WT 5-HT_2C_R protein and the GluN2A protein and further demonstrated that the F327L human mutation impairs this interaction. Consistently, we demonstrated that 5-HT/5-HT_2C_R signals facilitate NMDA currents in vCA1 neurons and the development of EPSC LTP, which are attenuated by the F327L mutation. Several previous studies have examined the role of other 5-HT receptors in mediating the effects of 5-HT on learning and memory. For example, 5-HT 4 receptors (5-HT_4_Rs) have been shown to mediate the potentiation of hippocampal CA3 inputs to the dorsal CA1 (*6*), and chronic stimulation of 5-HT_4_Rs with a partial agonist can prevent cognitive deficits in a 5XFAD transgenic Alzheimer’s disease (AD) mouse model (*7*). Inhibition of 5-HT 1A receptors can enhance learning and memory in animal models (*8*, *9*), and administration of 5-HT 6 receptor antagonists can improve cognitive performance in behavioral tests (*10*). Thus, serotonin may exert multiple effects on cognitive function by acting through different 5-HT receptors.

The impairments in memory observed in *Htr2c^F327L/Y^* mice highlight the essential role of 5-HT_2C_R signaling in cognitive function in humans. We identified these *HTR2C* variants by studying people with severe obesity and show that *Htr2c^F327L/Y^* mice develop hyperphagic obesity on a high-fat diet (*20*). It is unlikely that the impaired memory in *Htr2c^F327L/Y^* mice is secondary to obesity per se because we observed these memory deficits in chow-fed *Htr2c^F327L/Y^* mice with comparable body weight to their WT littermates. Similarly, all other genetic mouse models used in our study exhibit altered memory without changes in body weight. Further, a subthreshold dose of lorcaserin improves memory without changing body weight. We conclude that endogenous 5-HT_2C_R signals are required for the regulation of both memory and body weight through distinct 5-HT_2C_R–expressing neuronal populations in the hypothalamus and hippocampus (*31*, *34*, *42*, *43*). Mutations of the *HTR2C* gene in humans, which affect all cells, cause both obesity and memory deficits.

While there is no evidence yet linking *HTR2C* gene variants with AD, reduced brain 5-HT bioavailability has been observed in the brains of patients with AD. Several studies have reported a decreased number of 5-HT neurons (*44*–*49*) and reduced levels of 5-HT and its metabolites (*50*, *51*) in the brains of patients with AD. Consistently, in an App transgenic mouse model, amyloid-β depositions were found in the projection sites of 5-HT neurons, which lead to the degeneration of 5-HT axons followed by their cell bodies (*52*). Here, we found that chemogenetic stimulation of vCA1-projecting 5-HT neurons fails to improve memory in *App^NL-G-F^* mice, which is likely due to a substantial loss of 5-HTergic projections to the vCA1 observed in these mice. Lorcaserin (a selective 5-HT_2C_R agonist) can restore neural plasticity and memory in *App^NL-G-F^* mice, suggesting that despite the lack of 5-HTergic projections to provide the ligand (5-HT), the downstream 5-HT_2C_R–expressing neurons in the vCA1 of *App^NL-G-F^* mice may be relatively intact and can still respond to exogenous 5-HT_2C_R agonists. Of course, another possibility that we could not exclude is that lorcaserin may act upon other 5-HT_2C_R–expressing populations to improve cognition. Evidence suggests that 5-HT_2C_Rs expressed by the medial prefrontal cortex (*53*), the dentate gyrus (*17*), the bed nucleus of stria terminalis (*54*), and the basolateral amygdala (*55*) are involved in memory regulation.

## MATERIALS AND METHODS

### Human studies

These studies were approved by the Cambridge Local Research Ethics Committee (03/103, 03/104, 18/EE/0032) and conducted in accordance with the principles of the Declaration of Helsinki. Each participant provided written, informed consent. All participants completed the PRMQ that assesses the frequency of everyday memory performance failures. Items are rated on a 5-point Likert-type scale ranging from 1 (never) to 5 (very often). Raw scores were transformed into T scores using methods detailed previously (*21*).

### UK Biobank exome analysis

This research was conducted using the UK Biobank Resource under application 53821. We used pVCF variant file (chrX, block 17) from OQFE exome pipeline (UK Biobank Field 23148, OQFE 450K exomes interim release). We split and left-normalized multiallelic entries (bcftools v.1.9) and defined variant consequences with respect to Ensembl canonical transcript ENST00000276198 using Ensembl Variant Effect Predictor (Ensembl release v.109). Genetically determined sex was obtained from Field 31. In females, we used genotype calls as provided in the pVCF file. In males, we set to homozygous reference any reported heterozygous call (“0/1”) for which the reference allele read count was >0 (*N* = 63). Relatedness was obtained from the UK Biobank (ukbgene rel), and one person was excluded from each related pair among all the OQFE exomes (kinship ≥ 0.0442, KING, third-degree kinship or closer; retained pairs contained within OQFE exomes and excluded individuals in column “ID2”). European genetic ethnic grouping was obtained from Field 22006 (self-reported “white British” and tight cluster in genotype principal components analysis). Unrelated European exomes were taken forward for analysis (*N* = 147,346 males, *N* = 171,019 females). Prospective memory (PM) and fluid intelligence (FI) scores were available for participants to whom these tests had been presented at initial assessment center visit: PM (Field 20018; *N* = 49,477 males and *N* = 56,593 females were presented with the test in our study cohort) and FI (Field 20016; *N* = 48,441 males and *N* = 55,525 females began the puzzles in our study cohort). For each GOOS variant previously investigated for LOF in cells (*20*), the effect of carrier status on FI score and PM recall among male or female participants was estimated using Mann-Whitney *U* and Fisher’s exact test due to the imbalance of carriers and noncarriers of rare variants (table S2). The effect of T419A carrier status on FI score and the odds of correct recall (“Recalled”) among males was subsequently tested using regression with covariates age and age^2^ (table S2). The proportion of self-reported professional diagnoses of depression or anxiety (Field 20544) were subsequently inspected (table S2). We obtained self-reported professional diagnoses of “Depression” and “Anxiety, nerves or generalized anxiety disorder” from the Mental Health online follow-up questionnaire (described in https://biobank.ctsu.ox.ac.uk/showcase/refer.cgi?id=22) for which there was an available “Date of completing” (Field 20400) for *N* = 46,137 males and *N* = 58,220 females among our study cohort.

### Mice

Care of all animals and procedures were approved by the Baylor College of Medicine Institutional Animal Care and Use Committee. Multiple lines of transgenic mice were used in the current study. *Tph2-CreER* mice were purchased from the Jackson Laboratory (#016584), which express tamoxifen-inducible Cre recombinase selectively in 5-HT neurons, as we validated previously (*32*). These mice were used for neurotracing, CRACM, and chemogenetic studies as described below. A *Rosa26-LSL-tdTOMATO* allele (the Jackson Laboratory, #007905) (*56*) was crossed onto some *Tph2-CreER* mice for histology and neurotracing studies. *App^NL-G-F^* mice (*37*) were obtained from RIKEN, Japan. Here, we crossed heterozygous *App^NL-G-F^* mice to generate littermates of male WT and homozygous *App^NL-G-F^* mice. These mice were used in behavioral, electrophysiology, and Western blotting studies as described below. We also crossed to generate littermates of *App^NL-G-F^* and *App^NL-G-F^*/*Tph2-CreER* mice for chemogenetic experiments.

The *Htr2c^flox^* mouse allele was previously generated and validated in J. Elmquist’s laboratory (*31*). Note that the *Htr2c* gene is on the X chromosome, so male mice only have one copy, while females have two copies. Here, we crossed female heterozygous *Htr2c^floxl+^* mice with male C57Bl6j mice to generate littermates of male WT and hemizygous *Htr2c^flox/Y^* mice. These mice (4 months of age) received stereotaxic injections of AAV-Cre-GFP into both sides of the vCA1 [anterior-posterior (AP): −3 mm, medial-lateral (ML): +3 mm and −3 mm, and dorsal-ventral (DV): −4.5 mm]. After 9 weeks of recovery, at the age of 6 months, these mice were used in behavioral and electrophysiology studies, as described below. At the end of behavioral studies, mice were anesthetized and perfused transcardially with 0.9% saline followed by 10% formalin. Brains were removed, postfixed in 10% formalin for 16 hours at 4°C, and cryoprotected in 30% sucrose for 48 hours. Brains were frozen, sectioned at 14 μm using the cryostat, and washed in diethyl pyrocarbonate (DEPC)–treated phosphate-buffered saline (PBS) for 10 min. Sections were mounted on DEPC-treated charged slides, dried for 0.5 hours at room temperature, and stored at −80°C. On the day of the RNAscope assay, the slides were thawed and slides were rinsed two times in PBS 1× and placed in an oven for 30 min at 60°C. After that, slides were postfixed in 10% formalin for 15 min at 4°C. Slides were then gradually dehydrated in ethanol (50, 70, and 100%, 5 min each) and underwent target retrieval for 5 min at 100°C. Slides were incubated in protease III (#322337, ACDBio) for 30 min at 40°C. Slides were then rinsed in distilled water and incubated in RNAscope probes for *Htr2c* (Mm-Htr2c; #401001, ACDBio) for 2 hours at 40°C. Sections were then processed using the RNAscope Fluorescent Multiplex Detection Reagents (#320851, ACDBio) according to the manufacturer’s instructions. Slides were cover-slipped and analyzed using a fluorescence microscope.

*Tph2^flox^* mice were purchased from the Jackson Laboratory (#027590), which allow selective deletion of Tph2 in a Cre-dependent manner. Here, we crossed heterozygous *Tph2^flox/+^* mice and homozygous *Tph2^flox/flox^* mice to generate littermates of male *Tph2^flox/+^* and *Tph2^flox/flox^* mice. These mice (4 months of age) received stereotaxic injections of the retrograde AAV-Cre-GFP into both sides of the vCA1 (200 nl per side, AP: −3 mm, ML: +3 mm and −3 mm, and DV: −4.5 mm). After 9 weeks of recovery, at the age of 6 months, these mice were used in behavioral and electrophysiology studies as described. At the end of the experiment, all mice were perfused with 10% formalin. Brain sections (25 μm in thickness) were collected. The sections were blocked (3% normal goat serum) for 1;hour, incubated with rabbit anti-Tph2 antibody (1,3,000, Ab111828, Abcam) on a shaker at room temperature overnight, followed by goat anti-rabbit Alexa Fluor 405 (1,200, A31556, Invitrogen) for 2 ;hours. Slides were cover-slipped and analyzed using a fluorescence microscope. Neurons with Tph2 immunoreactivity in the DRN, MRN, and CRN were counted; three mice per group were included in this analysis.

The F327 amino acid residue in the human 5-HT_2C_R protein is equivalent to the F328 amino acid residue in the mouse 5-HT_2C_R protein. Here, we generated a knock-in mouse with the F328L mutation, referred to as *Htr2c^F327L^* for simplicity. Briefly, a single-guide RNA (sgRNA) (5′- TTTCATCACCAATATCCTGT) was purchased from Synthego (Menlo Park, CA) and used to target a double strand–breading site near the F328 codon of the mouse Ht2c gene. Cas9 Nuclease was purchased from IDT (Alt-R S.p. Cas9 Nuclease V3, Coralville, IA). The donor single-stranded DNA (ssDNA) template to introduce the F328L point mutation, as well as a silent mutation C326C to remove the restriction site for NlaIV, was purchased from IDT (Coralville, IA). The sequence of ssDNA is as follows: 5′-AATGAGAAGAAAGCTTCCAAAGTCCTTGGCATTGTATTCTTTGTGTTTCTG ATCATGTGGTGTCCGCTTTTCATCACCAATATCCTGTCGGTGCTTTGTGGGAAGGCCTGTAACCAAAAGCTAATGGAGAAACTTCTCAATGTGTTTGTTTGGATT. The BCM Genetically Engineered Murine Model (GERM) Core microinjected Cas9 (20 ng/μl), ssDNA (20 ng/μl), and sgRNA (20 ng/μl) into the pronuclei of 139 one-cell stage C57Bl/6J embryos. Pronuclear injections were performed using a microinjection needle (1 mm outer and 0.75 mm inner) with a tip diameter of 0.5 to 0.75 μm, an Eppendorf Femto Jet 4i to set pressure and time to control injection volume (0.5 to 1 pl per embryo). Injections were performed under a 200 to 400× magnification with Hoffman modulation contrast for visualizations. Founder animals (F_0_) were identified by polymerase chain reaction (PCR)–based restriction digestion to detect the CRISPR-generated point mutations in the Ht2c gene. PCR product was amplified with the primer pairs: 5′-ACGTCGAAAGAAGAAAGAAAAGC and 5′-GGTAAATTTTGTTGAAGAGAGTGTAC. The 266–base pair (bp) PCR products were then digested with NlaIV. After the digestion, 153-, 79-, and 34-bp fragments could be detected for PCR products from a WT allele; 232- and 34-bp fragments could be detected from a mutant *Htr2c^F327L^* allele. Three independent lines were sequenced for the further confirmation of the point mutation. One of these lines was crossed to C57Bl/6j to produce study cohorts. Potential off-target sites were predicted by Cas-OFFinder, and genomic sequence containing these sites were PCR-amplified and sequenced; no off-target mutation was identified in all these regions. Female heterozygous *Htr2c^F327L/+^* mice were crossed with male C57Bl/6j to generate littermates of male WT and hemizygous *Htr2c^F327L/Y^* mice, which were used in behavioral and electrophysiology studies as described below.

All the breeders have been backcrossed to C57Bl6j background for more than 12 generations. Mice were housed in a temperature-controlled environment in groups of two to five at 22° to 24°C using a 12-hour light/12-hour dark cycle. All mice were fed standard chow (6.5% fat, #2920, Harlan-Teklad, Madison, WI) ad libitum, unless described otherwise. Water was provided ad libitum.

### Coimmunoprecipitation in cells and mouse brains

HEK293T cells were cultured in American Type Culture Collection–formulated Eagle’s minimum essential medium supplemented with 10% (v/v) of fetal bovine serum. The constructs of *Htr2c^WT^* and *Htr2c^F327L^* were generated and sequenced previously (*20*). Cells were then transiently transfected with indicated constructs using Lipofectamine 3000 reagent (L3000015, Invitrogen). Typically, 500 ng of plasmid DNA were transfected to each well of the 24-well plate. The amount of plasmid DNA was scaled up and down on the basis of the growth surface area. All experiments were performed 48 hours after transfection. To identify the interaction between Htr2c and GluN2A in vitro, HEK293T cells were cotransfected with *Htr2c^WT^* (or *Htr2c^F327L^*) and GluN2A-GFP constructs. Forty-eight hours after transfection, the cells were lysed with NP-40 buffer supplemented with protease and phosphatase inhibitors. Then, the cell lysates were incubated with anti-GFP magnetic beads (gtma, Proteintech) for 1 hour at 4°C with gentle rocking. The beads were washed four times with NP-40 buffer. After the final wash, the protein complex was eluted from the beads by adding 2XSDS buffer and boiling at 95°C for 10 min.

To confirm the interaction between Htr2c and GluN2A in vivo, the vCA1 of WT and *Htr2c^F327L/Y^* mice (2 months of age) were dissected, and the tissues were homogenized by sonication in NP-40 buffer. The cell lysates were incubated with primary antibody against GluN2A (4205S, Cell Signaling Technology) overnight at 4°C with gentle rocking. After the incubation, the Protein A/G magnetic beads (88802, Pierce) were added to the lysate-antibody mixture and incubated for 1 hour. The beads will bind to the primary antibody along with any interacting proteins. Afterward, the beads were washed four times with NP-40 buffer. After the final wash, the protein complex was eluted from the beads by adding 2XSDS buffer and boiling at 95°C for 10 min. Western blotting was used to analyze the eluted proteins with primary antibodies against Htr2c (sc-17797, Santa Cruz Biotechnology), GluN2A (4205S, Cell Signaling Technology), and GFP (2956S, Cell Signaling Technology).

### Immunohistochemistry

*Tph2-CreER/Rosa26-LSL-tdTOMATO* and *App^NL-G-F^/Tph2-CreER/Rosa26-LSL-tdTOMATO* mice receive tamoxifen induction at 8 weeks of age. At 6 months of age, these mice were transcardially perfused with saline, followed by 10% formalin. The brain sections were cut at 25 μm and collected into five consecutive series. One series of the sections was mounted on charged slides, and tdTOMATO-labeled cell bodies in the DRN and MRN, as well as fibers/terminals in the vCA1, were assessed under a fluorescence microscope. ImageJ was used to quantify the fluorescence density, and three mice were included in each group for statistical analyses.

### Behavioral tests

All behavioral tests were conducted by the same person between 9 a.m. and 4 p.m. The experimenter was blinded to genetic information of each test subject. Two different memory function tests were conducted to evaluate cognitive functions. The 6-arm RAWM test was performed first, followed by fear conditioning test 2 days after the completion of RAWM. To avoid potential confounding effects of divergent body weight on the performance of mice in these tests, we only used chow-fed mice with matched body weight. In the lorcaserin study, we selected a subthreshold dose of lorcaserin, which did not change the animals’ body weight during the course of treatment and behavioral tests.

#### RAWM

The apparatus of RAWM consists of a plastic pool that is made from FDA-approved material, with an internal diameter of 0.9 m and depth of at least 25 cm. To add spatial complexity, we insert six arms that are made of aluminum. The inserts are placed in a circular pool filled with lukewarm water that sufficiently hides the platform. The aluminum inserts are 20 cm high and 33 cm of length. The arms are arranged such that the passages are equally spaced, with 60° of separation angles. The central area is approximately 36 cm in diameter. The passage width is 20 cm, and the escape platform has a diameter 10 cm. During the learning trials, one mouse was released from a random arm. The time the mouse used before it found the escape platform was recorded. If a mouse takes longer than 1 min to find the platform, we note the latency as 60 s. When the mice consistently made less than two errors, they were considered to have learned where the platform was located. In this study, all mice received 12 learning trials. If a mouse constantly takes longer than 1 min to find the platform in 12 learning trials, it will be excluded from the test group. For the short-term memory, mice were tested in the same apparatus 30 min after the last learning session; for the long-term memory, mice were tested again 24 hours later.

#### Fear conditioning

Each mouse was placed in the fear conditioning box (Omnitech Electronics) for 5 min before training to acclimatize to the environment. The chamber was well ventilated, and the walls and top were clear acrylics for easy recording. The flooring consisted of stainless steel rods above a removable waste tray, which was cleaned with water and a paper towel between tests and subjects. The stimulus hub, which delivers the sound and light cues, is located at the top. The training started with a 90-s habituation period, followed by 10 cues (CS) and foot shock (US) cycles, with a 2-s interval between CS and US and a 2-min interval between each cycle. The US consists of a 20-s light cue (128-cd intensity) and sound cue (225-W/m^2^ intensity, 10,000 Hz). Subjects were returned to their home cage after training.

Contextual memory (day 2): Twenty-four hours after training, the subject was placed back into the original context to examine contextual memory. The test was performed for the period of time that equals to three cycles of US-CS but without the presentation of US. After the contextual memory test, the subject was returned to its home cage.

Tracing memory (day 3): Twenty-four hours after the contextual memory test, the subject was placed into the fear conditioning box with a different context (different color and scent). Three cycles of CS were delivered without US. The subject was returned to its home cage and waited for further tests.

#### Hot plate test

Mice were transported to the study room 1 hour before the test to acclimatize. Mice were placed individually onto a hot plate analgesia meter (Panlab) that was set at 52°C. We recorded how long it took for the mouse to show paw shaking, licking, or jumping. If the mouse did not show these behaviors in 30 s, we removed the mouse, and 30 s was considered as latency by default. Mice were returned to their home cage after tests.

#### Open field test

We used SuperFlex Open Field system (Omnitech Electronis, Inc. Columbus, Ohio) to monitor the locomotor activity and anxiety-like behavior in mice. Mice were habituated to the test room 3 days before the test. On the test day, in dim light (100 to 200 lux), the mouse was placed into the center of the 40.64 cm–by–40.64 cm acrylic arena with photosensors that create a 16 × 16 infrared grid. The mouse’s movement interferes with the infrared beams, and the interferences were recorded during the 15-min test period. After the test, mice were returned to their home cage. Results were analyzed by the Fusion software (Omnitech Electronics Inc. Columbus, Ohio). During analysis, the arena was divided into center zone (40% of the total surface area of the arena at the center) and margin zone (the remaining surface area at the edges of the arena). The activity of each mouse in the two zones was measured.

#### Fiber photometry

To evaluate how the activities of 5-HT_2c_R–expressing vCA1 neurons correlate to cognitive behaviors, we recorded calcium activities of vCA1 neurons in male *Htr2c^F327L/Y^* mice and their WT littermate mice during fear memory test. Male *Htr2c^F327L/Y^* mice and their WT littermate mice (5 months of age) were anesthetized by isoflurane and received stereotaxic injections of retrograde pAAV-Syn-GCaMP6f-WPRE-SV40 (#100837-AAV9; Addgene) into the vCA1. During the same surgery, an optical fiber (fiber: core = 400 μm, 0.48 NA, M3 thread titanium receptacle; Doric Lenses) was implanted over the vCA1. Fibers were fixed to the skull using dental acrylic, followed by a 4-week recovery. All mice were allowed to adapt to the tethered patchcord for 2 days before experiments and given 5 ;min to acclimate to the tethered patchcord before recording during the fearing conditioning. For each recording, continuous <20-μW blue light-emitting diode (LED) at 465 nm and ultraviolet (UV) LED at 405 nm served as excitation light sources, driven by a multichannel hub (Doric Lenses), modulated at 211 ;and 330 Hz, respectively. The light was delivered to a filtered minicube (FMC5, Doric Lenses) before connecting through optic fibers to a rotary joint (FRJ 1 × 1, Doric Lenses) to allow free movement. GCaMP6 calcium GFP signals and UV autofluorescent signals were collected through the same fibers back to the dichroic ports of the minicube into a femtowatt silicon photoreceiver (2151, Newport). The digital signals were then amplified, demodulated, and collected through a lock-in amplifier (RZ5P, Tucker-Davis Technologies). The fiber photometry data were downsampled to 8 Hz. We aligned acute neural responses (five readings/s) to every cue and foot shock/predicted foot shock. We derived the values of fluorescence change (Δ*F*/*F*_n_) by calculating (*F*_465_ − *F*_405_) / *F*_405_ to minimize the interference of movement and/or bleaching artifacts.

### Chemogenetics

Male *Tph2-CreER* mice and WT littermate mice (at 5 months of age) were anesthetized by isoflurane and received bilateral stereotaxic injections of the retrograde pAAV-DIO-hM4D(Gi)-mCherry into the vCA1 region. Similarly, male *App^NL-G-F^/Tph2-CreER* mice and *App^NL-G-F^* littermate mice (at 5 months of age) were anesthetized by isoflurane and received bilateral stereotaxic injections of the retrograde pAAV-DIO-hM3D(Gq)-mCherry into the vCA1 region. Tamoxifen [0.2 mg/g, intraperitoneally (ip)] was injected into each mouse to induce Cre recombinase activity, followed by a 4-week recovery. All the mice were then subjected to RAWM and fear conditioning tests when vCA1-projecting *Tph2*-expressing neurons were chemogenetically inhibited or activated with CNO injections (1 mg per kg, ip). CNO was injected into each mouse 30 min before RAWM and fear conditioning training, 6 hours after training, and 30 min before the tests. To prove that CNO does inhibit/activate vCA1-projecting *Tph2*-projecting neurons, we used some mice for in vitro electrophysiology to measure responses in firing frequency and RM potential in response to 10 μM CNO treatment.

### Lorcaserin treatment

Male *App^NL-G-F^* mice and WT littermates received treatment of vehicle or lorcaserin (ip, 1 mg per kg body weight per day for 2 weeks) starting at the age of 5 months and 3 weeks. Then, these mice continued to receive daily treatment when they were subjected to the behavioral tests described above. The treatments were given every day at 5:00 p.m. till the last day of behavioral tests.

### Home cage scan

Mice were treated with lorcaserin (1 mg/kg) or saline at 5 p.m. each day for 14 consecutive days. The mice were singly housed for the second 7 days before the test. Then, their home cages were put into HomeCageScan chambers (Cleversys Inc.) at 6 p.m. to record their sleeping patterns during a 24-hour period. Total sleep time during the light/dark cycle was calculated for each mouse.

### Electrophysiology

Mice (males, 6 to 8 months) were anesthetized with isoflurane and were transcardially perfused with a modified ice-cold sucrose-based cutting solution (pH 7.4; containing 10 mM NaCl, 25 mM NaHCO_3_, 195 mM sucrose, 5 mM glucose, 2.5 mM KCl, 1.25 mM NaH_2_PO_4_, 2 mM sodium pyruvate, 0.5 mM CaCl_2_, and 7 mM MgCl_2_, bubbled continuously with 95% O_2_ and 5% CO_2_). The mice were then decapitated, and the entire brain was removed and immediately submerged in the cutting solution. Coronal slices (250 μm) were cut with a Microm HM 650 V vibratome (Thermo Fisher Scientific). Brain slices containing the vCA1 were collected for each corresponding mouse, and recordings were made at levels throughout this brain region. The slices were recovered for ~30 min at 32°C and then maintained at room temperature for another 1 hour in oxygenated (95% O_2_ and 5% CO_2_) artificial cerebrospinal fluid (ACSF, pH 7.4; containing 126 mM NaCl, 2.5 mM KCl, 2.4 mM CaCl_2_, 1.2 mM NaH_2_PO_4_, 1.2 mM MgCl_2_, 11.1 mM glucose, and 21.4 mM NaHCO_3_) before recording.

Slices were transferred to the recording chamber at 32°C and perfused continuously with oxygenated ACSF at a flow rate of 1.8 to 2.0 ml/min. Slices were allowed to equilibrate for at least 5 min before recording. Pyramidal neurons in vCA1, identified on the basis of their location and morphology, were visualized using epifluorescence and infrared–differential interference contrast imaging on an upright microscope (Eclipse FN-1, Nikon) equipped with a moveable stage (MP-285, Sutter Instrument). Recordings were made using a MultiClamp 700B amplifier (Axon Instruments), sampled using Digidata 1440A, and analyzed offline with pClamp 10.3 software (Axon Instruments). Series resistance was monitored during the recordings. The liquid junction potential was monitored and corrected. Data were excluded if the series resistance exceeded a 20% change during the experiment. Recordings were amplified, filtered at 1 kHz, and digitized at 10 kHz.

To record action firing potential in response to lorcaserin puff, patch pipettes with resistances of 3 to 5 MΩω were filled with an intracellular solution (pH 7.3) containing 128 mM potassium gluconate, 10 mM KCl, 10 mM Hepes, 0.1 mM EGTA, 2 mM MgCl_2_, 0.05 mM guanosine triphosphate (GTP) (sodium salt), and 4 mM adenosine triphosphate (ATP) (magnesium salt). Current clamp was engaged to test neural firing frequency and RM potential at the baseline and after puff delivery of lorcaserin (100 μM, 5 s). Each neuron received a small amount of current injection to maintain the RM close to the spontaneous firing threshold (~ −55 mV) before the treatment. To ensure that each recorded neuron receives the same amount of lorcaserin, the neurons located on the surface of the slice were selected to record and the puff pipette was always put at a 100-μm horizontal and 100-μm vertical distance from the recorded neurons. The puff strength was maintained at the same level using a repeatable pressure pulse system (Picospritzer III, Parker). Each neuron was recorded for at least 1 min at baseline, and only the neurons with a stable baseline were used to test the lorcaserin treatment. The values of RM and firing frequency were averaged at baseline and in a 1-min range containing the point with the maximal change in RM potential after lorcaserin puff. A neuron was considered depolarized or hyperpolarized if a change in membrane potential was ≥2 mV, whereas values between 2 mV was defined as “irresponsive.”

To record AMPA current, patch pipettes with resistances of 2 to 3 MΩω were filled with an intracellular solution (pH 7.3) containing 100 mM CsCH3SO3, 20 mM KCl, 10 mM Hepes, 4 mM Mg-ATP, 0.3 mM GTP (sodium salt), 7 mM tris2-phosphocreatine, and 3 mM QX-314. The membrane potential was held at −70 mV in the voltage-clamp mode, and evoked EPSCs (eEPSCs) were elicited in the presence of 20 μM bicuculline using a bipolar stimulating electrode (CBARC75, FHC Inc.) placed in the ventral stratum radiatum 300 μm away from the recording site. The rectangle current pulses (duration: 0.2 ms, frequency: 0.05 s^−1^) were delivered via a constant-current stimulator (ISO-FLEX, Microprobes). A series of stimulation intensities ranging from 0 to 200 μA with a step of 20 μA was delivered to elicit the maximum magnitude of the EPSCs. To record the EPSC-based LTP, the stimulation intensity was adjusted to elicit an EPSC with an amplitude 30 to 50% of the maximum. Baseline eEPSCs were recorded for 5 min (response variability <10%). After baseline recordings, two trains of high-frequency stimulation (100 pulses at 100 s^−1^) separated by 20s were delivered to induce LTP. In the LTP saturation recordings, additional three pairs of high-frequency stimulations were delivered with a 10-min interval between each pair. To record NMDA currents, the membrane potential was held at +40 mV in the voltage-clamp mode. The GABA_A_ receptor antagonist, bicuculline (20 μM), and the α-amino-3-hydroxy-5-methylisoxazole-4-propionic acid and kainic acid (AMPA/KA) receptor antagonist, DNQX (20 μM), were added in the ACSF to isolate the NMDA component in the eEPSCs. The stimulation intensity from 0 to 200 μA with a 20-μA gradient was applied to eliminate the impact caused by the unequal stimulation intensity received by the recorded neuron.

### Channel rhodopsin–assisted circuit mapping

CRACM was used to establish a functional connection between 5-HT^DRN^ neurons and vCA1 neurons. Specifically, 8-week-old *Tph2-CreER* mice were injected with 200-nl AAV8-EF1α-DIO-hChR2(H134R)-EYFP into the DRN (AP: −4.65 mm, ML: 0 mm, and DV: −3.6 mm and −3.3 mm). The mice also received a tamoxifen injection 3 days after virus delivery (0.2 mg/g body weight, ip). Three weeks after injections, mice were euthanized and brain slices containing vCA1 neurons were prepared for electrophysiology. The whole-cell patch current clamp recordings were performed on vCA1 neurons in the brain slice to determine the responses of neural firing rate and membrane potential to the photostimulation of 5-HT^DRN^ neural fibers (473 nM, 40 mW, 10 ms pulse, 10 Hz for 2 min) in the presence of CNQX (30 μM), D-AP5 (30 μM), and bicuculline (50 μM) to minimize glutamatergic and GABAergic inputs. Then, the light-induced responses were tested again in the same neurons in the presence of the selective 5-HT_2C_R receptor antagonist, SB242084 (100 μM). In addition, the voltage clamp recordings were also performed to assess the eEPSC and eIPSC in vCA1 neurons in response to blue light pulses (473 nM, 40 mW, 10 ms pulse). Tetrodotoxin (1 μM) and 4-Aminopyridine (400 μM, 4-AP, a potassium channel blocker) were used for the validation of monosynaptic input in the recorded neurons. For light eEPSC recordings, the intracellular solution contained the following (in mM): 125 CsCH_3_SO_3_; 10 CsCl; 5 NaCl; 2 MgCl_2_; 1 EGTA; 10 Hepes; 5 (Mg)ATP; and 0.3 (Na)GTP (pH 7.30 with CsOH). The eEPSCs were recorded in whole-cell voltage-clamp mode by holding the membrane potential at Vh = −60 mV. Light-evoked EPSCs were reexamined in the same neurons in the presence of D-AP5 (30 μM) and CNQX (30 μM). For recording of eIPSCs, glass pipettes were filled with internal solution containing the following (in mM): 140 CsCl, 10 Hepes, 5 MgCl_2_, 1 BAPTA, 2 (Mg)ATP, and 0.3 (Na)2GTP (pH 7.30 adjusted with CsOH). The eIPSCs were recorded in whole-cell voltage-clamp mode by holding the membrane potential at Vh = −60 mV. eIPSCs were reexamined in the same neurons in the presence of bicuculline (50 μM).

### Neurotracing

To determine whether vCA1 neurons receive innervation from 5-HT neurons, 12-week-old WT mice were anesthetized by isoflurane and received stereotaxic injections of a retrograde virus expressing GFP (AAV.hSyn.eGFP-Cre, 1.3E + 13 GC/ml, #105540-AAVrg, Addgene) into the vCA1 (200 nl per side; AP: −3 mm, ML: +3 mm and −3 mm, and DV: −4.5 mm). Ten days after injections, mice were perfused with 10% formalin and brain sections were cut at 30 μm (five series). GFP expression was checked in the vCA1, DRN, MRN, and CRN. These brain sections were subjected to immunofluorescence staining for 5-HT with goat anti–5-HT antibody (1:3000, #20079, Immunostar) overnight, followed by the donkey anti-goat Alexa Fluor 594 (1:250, #110382, Jackson ImmunoResearch) for 2 hours. Slides were cover-slipped and analyzed using a Leica DM5500 fluorescence microscope with OptiGrid structured illumination configuration. The numbers of neurons that were 5-HT positive only or labeled by both 5-HT and GFP were counted in the DRN and MRN. The same experiment was repeated in three different mice for statistical analysis.

To further confirm the connections between 5-HT^DRN^ neurons (or 5-HT^MRN^ neurons) and vCA1, we generated a Cre-dependent AAV vector expressing WGA-GFP fusion protein (AAV-DIO-WGA-GFP). Briefly, WGA was subcloned from WGA-D (*57*) (#17989; Addgene), and the WGA-GFP (with a 27-bp short linker) was cloned into the Cre-dependent AAV vector (*58*). *Tph2-CreER/Rosa26-LSL-tdTOMATO* mice (12 weeks of age) were anesthetized by isoflurane and received stereotaxic injections of AAV-DIO-WGA-GFP virus (5.2E + 11 GC/ml) in the DRN (400 nl; AP: −4.65 mm, ML: 0 mm, and DV: −3.6 mm and −3.3 mm) or in the MRN (200 nl; AP: −4.65 mm, ML: 0 mm, and DV: −4.5 mm). These mice also received tamoxifen (0.2 mg/g body weight, ip) to induce Cre activity. After 4 weeks, mice were perfused as described above and brain samples were cut into 30-μm sections to check for GFP and tdTOMATO expression in the DRN, MRN, and/or vCA1.

### Statistics

The minimal sample size was predetermined by the nature of experiments. For most of the behavioral readouts, at least eight different mice per group were included. For electrophysiology studies, the same experiment was repeated in at least three different mice with multiple neurons. For neurotracing studies, at least three different mice were included. The data are presented as means ± SEM or as individual data points. Statistical analyses were performed using GraphPad Prism to evaluate normal distribution and variations within and among groups. Methods of statistical analyses were chosen on the basis of the design of each experiment and are indicated in figure legends. *P* < 0.05 was considered to be statistically significant.
