# Supplementary material for: Neural circuits expressing the serotonin 2C receptor regulate memory in mice and humans
Source: Sci Adv. 2024 Jun 28;10(26):eadl2675. doi: 10.1126/sciadv.adl2675 (PMC11212768; doi:10.1126/sciadv.adl2675)
Supplement: Supplementary file 1 — Figs. S1 to S8 Tables S1 and S2 [file sciadv.adl2675_sm.pdf]

Science Advances
AAAS

Supplementary Materials for

Neural circuits expressing the serotonin 2C receptor regulate memory in mice and humans

Hesong Liu *et al.*

Corresponding author: yongx@bcm.edu; isf20@cam.ac.uk

Sci. Adv. **10**, eadl2675 (2024)
DOI: 10.1126/sciadv.adl2675

This PDF file includes:

Figs. S1 to S8
Tables S1 and S2

Supplementary Figures

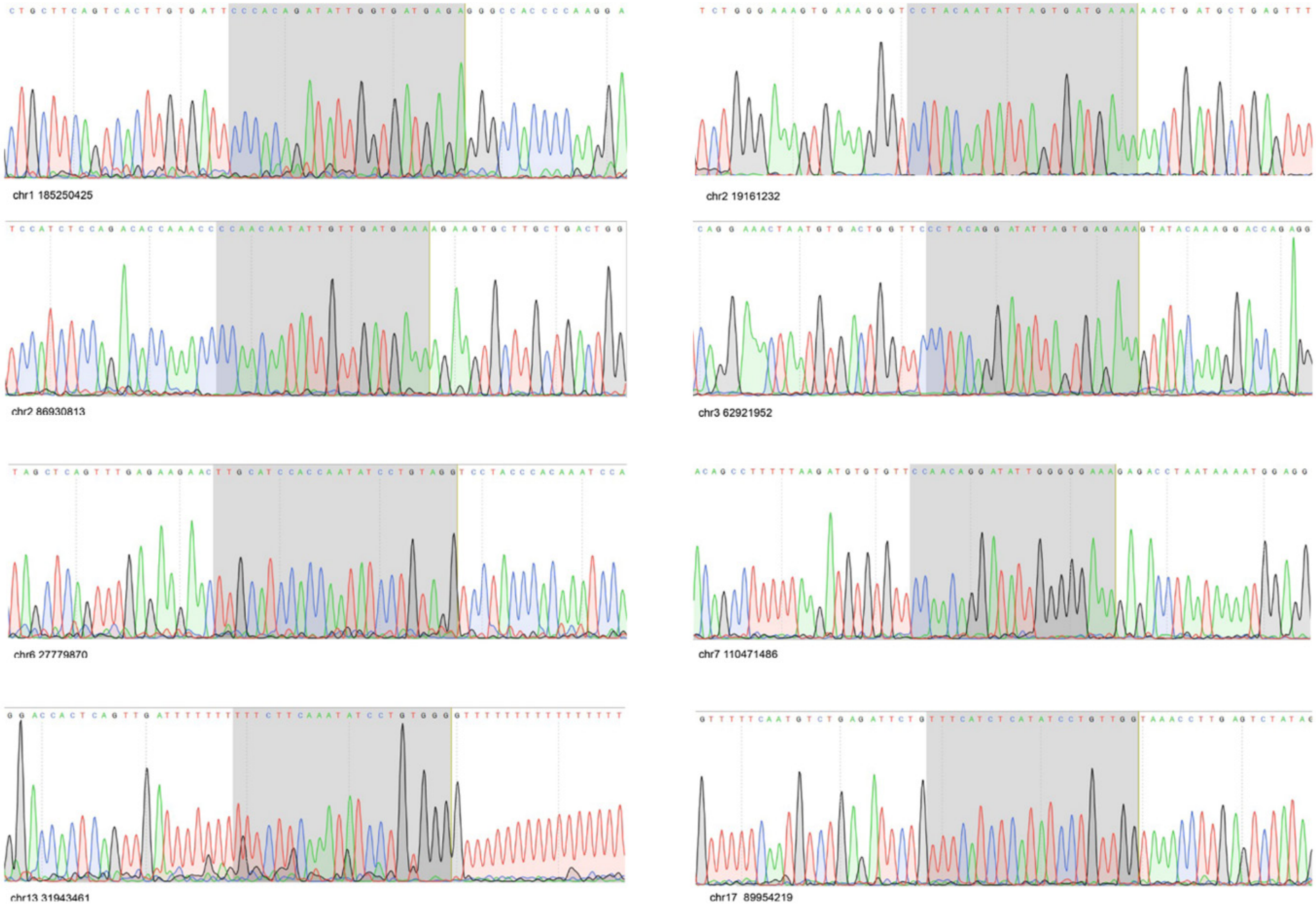

Fig. S1. No off-target mutation in *Htr2c*^{F327L} mice. Genomic sequence containing the potential off-target sites identified by Cas-OFFinder were PCR-amplified and sequenced. The grey highlight in each representative sequence trace is the region with potential off-target, but no mutation was identified in all these regions. Related to Fig. 1.

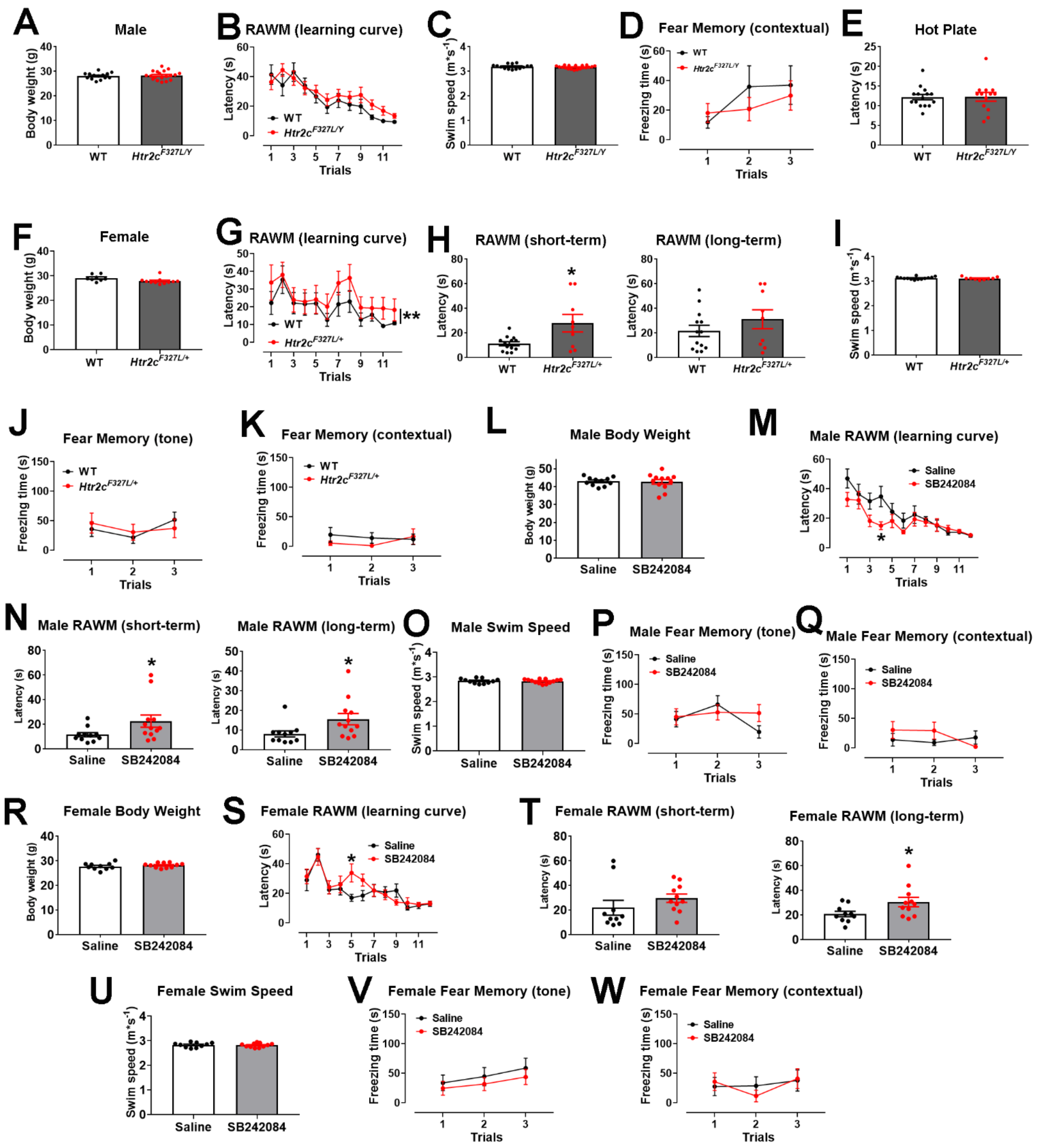

Fig. S2. 5-HT2CR regulates memory. (A) Body weight of male WT and *Htr2c*$^{F327L/Y}$ mice (6 months) at the time of memory tests. Results shown as mean ± SEM with individual data points (N=14 or 19 mice). **(B)** Latency to reach the platform during the 12 RAWM training trials. Results shown as mean ± SEM. **(C)** Swim speed during the RAWM. Results shown as mean ± SEM with individual data points. **(D)** Performance in the fear conditioning test reported as freezing time in response to contextual cues. Results shown as mean ± SEM. **(E)** Latency to lick hind paw or jump of male WT and *Htr2c*$^{F327L/Y}$ mice (6 months) in the hot plate test. Results shown as mean ± SEM with individual data points (N=13 or 15 mice). **(F)** Body weight of female WT and *Htr2c*$^{F327L/+}$ mice (6 months) at the time of memory tests. Results shown as mean ± SEM with individual data points (N=8 or 11 mice). **(G)** Latency to reach the platform

during the 12 RAWM training trials. Results shown as mean ± SEM. **, P<0.01 in two-way ANOVA analysis. **(H)** Latency to reach the platform in the RAWM test that took place 30 min (short-term) or 24 hr (long-term) after the last training session. Results shown as mean ± SEM with individual data points. *, P<0.05 in Mann-Whitney test as data were not normally distributed. **(I)** Swim speed during the RAWM. Results shown as mean ± SEM with individual data points. **(J-K)** Freezing time during the fear memory test in response to tone **(J)** or contextual **(K)** cues. Results shown as mean ± SEM. **(L)** Body weight of saline or SB242084-treated male WT mice (6 months) at the time of memory tests. Results shown as mean ± SEM with individual data points (N=11 or 12 mice). **(M)** Latency to reach the platform during the 12 RAWM training trials. Results shown as mean ± SEM. *, P<0.05 in two-way ANOVA analysis followed by Sidak tests. **(N)** Latency to reach the platform in the RAWM test that took place 30 min (short-term) or 24 hr (long-term) after the last training session. Results shown as mean ± SEM with individual data points. *, P<0.05 in Mann-Whitney test as data were not normally distributed. **(O)** Swim speed during the RAWM. Results shown as mean ± SEM with individual data points. **(P-Q)** Freezing time during the fear memory test in response to tone **(P)** or contextual **(Q)** cues. **(R)** Body weight of saline or SB242084-treated male WT mice (3 months) at the time of memory tests. Results shown as mean ± SEM with individual data points (N=10 or 11 mice). **(S)** Latency to reach the platform during the 12 RAWM training trials. Results shown as mean ± SEM. *, P<0.05 in two-way ANOVA analysis followed by Sidak tests. **(T)** Latency to reach the platform in the RAWM test that took place 30 min (short-term) or 24 hr (long-term) after the last training session. Results shown as mean ± SEM with individual data points. *, P<0.05 in two-sided unpaired t-test. **(U)** Swim speed during the RAWM. Results shown as mean ± SEM with individual data points. **(V-W)** Freezing time during the fear memory test in response to tone **(V)** or contextual **(W)** cues. Related to Fig. 1.

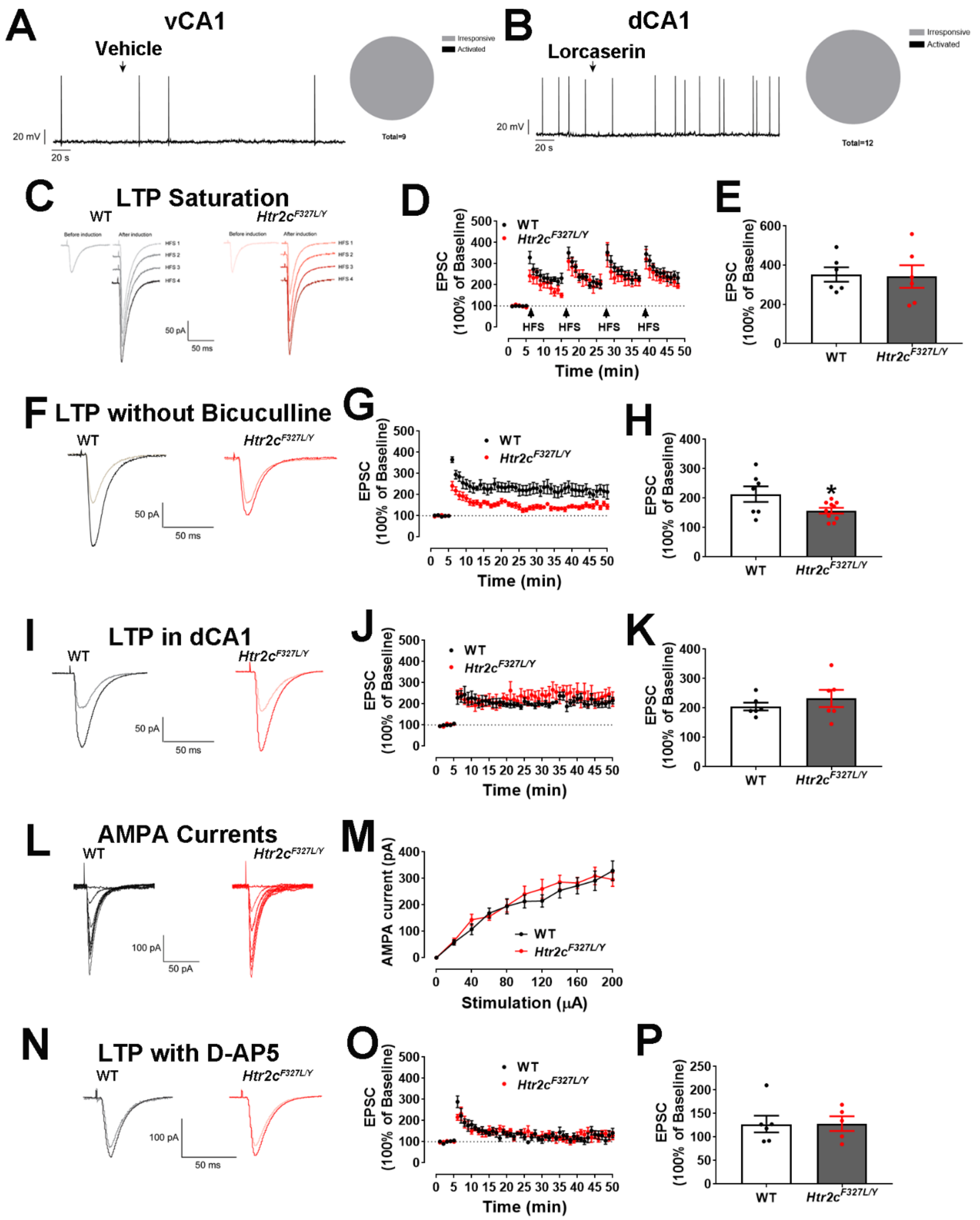

Fig. S3. 5-HT2CR regulates synaptic plasticity in vCA1 neurons. (A) Left panel: Representative current clamp traces in vCA1 neurons from WT mice treated with vehicle (5 seconds puff). Right panel: Percentage of neurons activated by vehicle puff or were irresponsive. (N= 9 neurons from 3 mice). **(B)** Left panel: Representative current clamp traces in dCA1 neurons from WT mice treated with lorcaserin (100 µM, 5 seconds puff). Right panel: Percentage of neurons activated by lorcaserin puff or were irresponsive. (N= 12 neurons from 3 mice). **(C)** Representative EPSC traces before and after LTP induction (with 4 HFS) in vCA1 neurons from WT or *Htr2c*$^{F327L/Y}$ mice. **(D)** Magnitude of EPSC elevations before and after LTP induction. **(E)** Averaged EPSC elevations in the first minute after 3rd pair of high-frequency stimulation in **(D)**. Results shown as mean ± SEM with individual data points (N=

6 neurons from 3 mice per group). **(F)** Representative EPSC traces before and after LTP induction in the absence bicuculline in vCA1 neurons from WT or *Htr2c*$^{F327L/Y}$ mice. **(G)** Magnitude of EPSC elevations before and after LTP induction. **(H)** Averaged EPSC elevations during 45-50 min in panel **(G)**. Results shown as mean ± SEM with individual data points. *, $P<0.05$ in two-sided unpaired t-test (N= 7 or 10 neurons from 3 mice per group). **(I)** Representative EPSC traces before and after LTP induction in dCA1 neurons from WT or *Htr2c*$^{F327L/Y}$ mice. **(J)** Magnitude of EPSC elevations before and after LTP induction. **(K)** Averaged EPSC elevations during 45-50 min in panel **(J)**. Results shown as mean ± SEM with individual data points (N= 6 neurons from 3 mice per group). **(L)** Representative AMPA current traces in vCA1 neurons from WT or *Htr2c*$^{F327L/Y}$ mice. **(M)** Magnitude of AMPA currents at different stimulations. Results shown as mean ± SEM (N= 7 or 10 neurons from 3 mice per group). **(N)** Representative EPSC traces before and after LTP induction in the presence of D-AP5 (50 μM) in vCA1 neurons from WT or *Htr2c*$^{F327L/Y}$ mice. **(O)** Magnitude of EPSC elevations before and after LTP induction. **(P)** Averaged EPSC elevations during 45-50 min in panel **(O)**. Results shown as mean ± SEM with individual data points (N= 5 or 6 neurons from 3 mice per group). Related to Fig. 1.

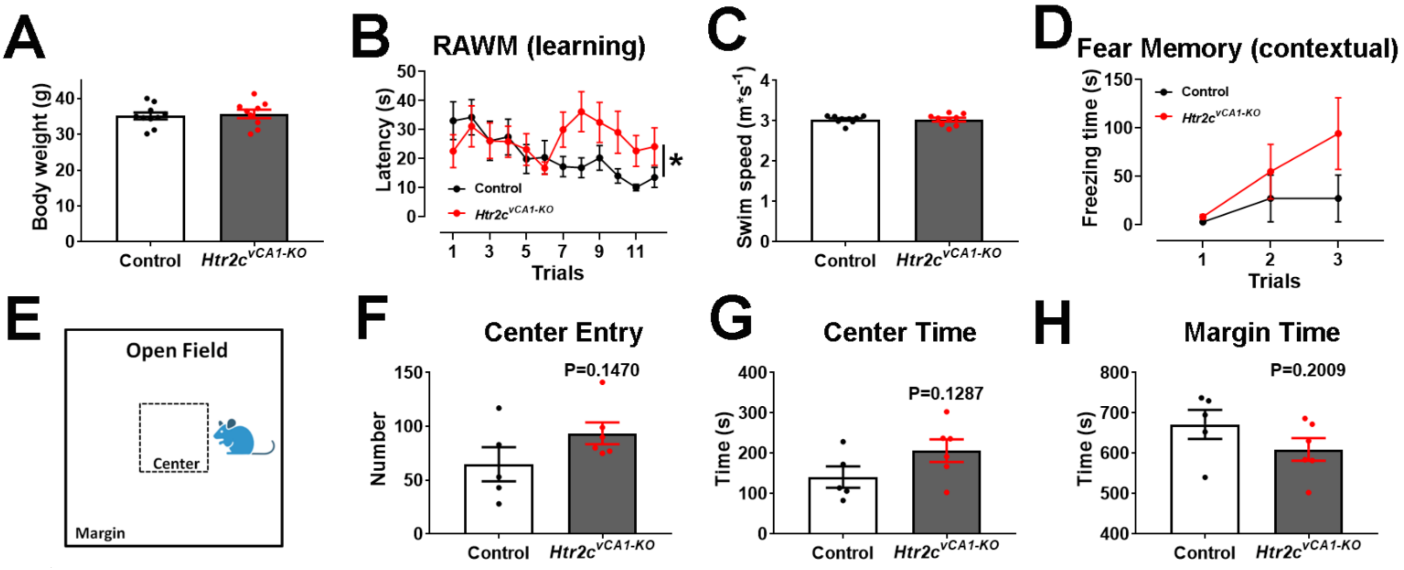

Fig. S4. 5-HT$_{2C}$R in the vCA1 regulates memory. (A) Body weight of male control and *Htr2c*$^{vCA1\text{-}KO}$ mice (6 months) at the time of memory tests. Results shown as mean ± SEM with individual data points (N=9 or 10 mice). **(B)** Latency to reach the platform during the 12 RAWM training trials. Results shown as mean ± SEM. *, P<0.05 in two-way ANOVA analysis. **(C)** Swim speed during the RAWM. Results shown as mean ± SEM with individual data points. **(D)** Performance in the fear conditioning test reported as freezing time in response to contextual cues. Results shown as mean ± SEM. **(E)** A schematic illustration of the open field test. **(F)** Number of entries to the center of the open field. Results shown as mean ± SEM. **(G)** Time spent in the center of the open field. Results shown as mean ± SEM. **(H)** Time spent in the margin of the open field. Results shown as mean ± SEM. Related to Fig. 2.

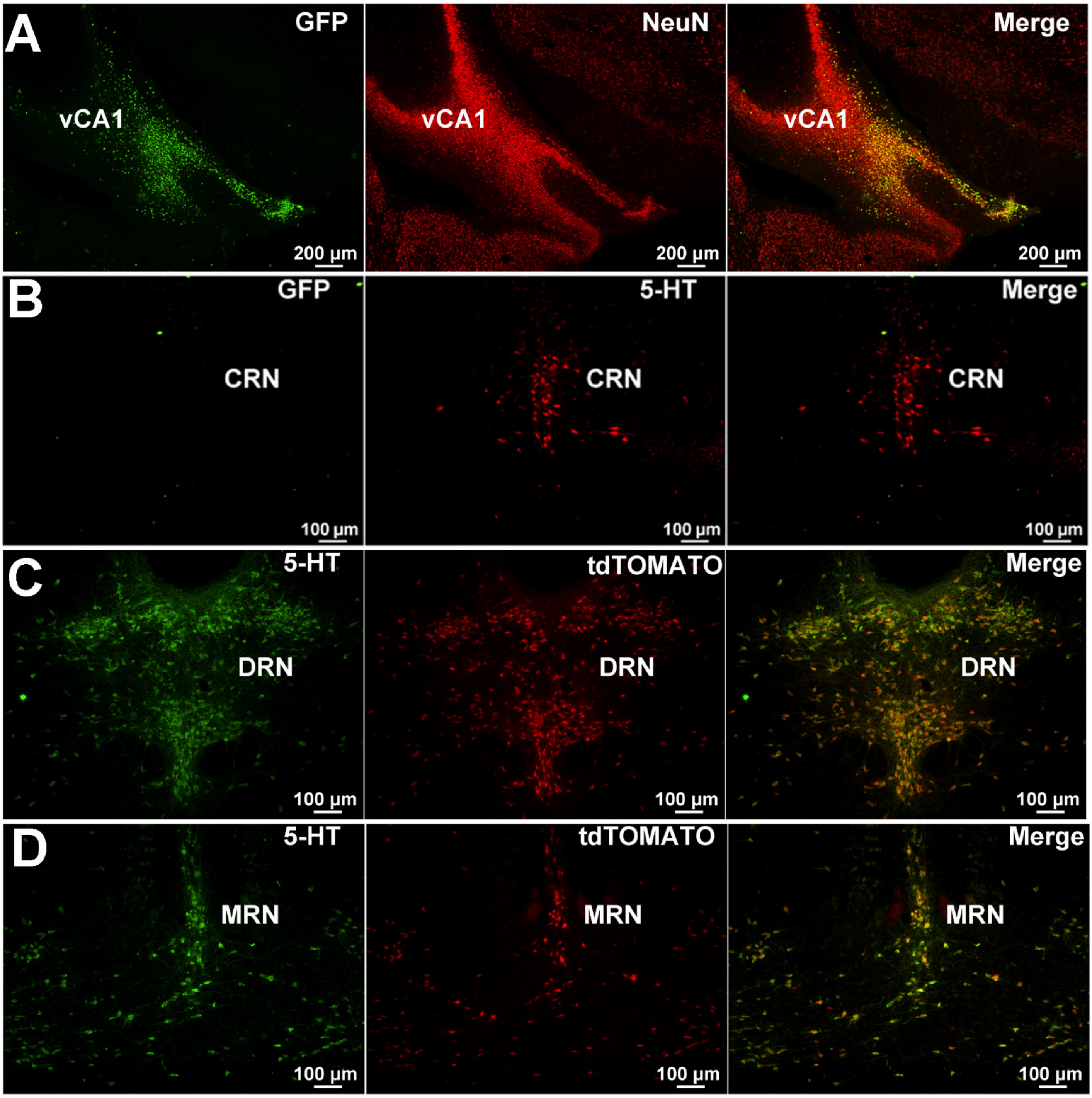

Fig. S5. Midbrain 5-HT neurons project to and activate vCA1 neurons. (A) Representative microscopic images showing GFP (left), NeuN-HT (middle) and merge (right) in the vCA1 of mice receiving retrograde AAV-Cre-GFP virus injected into the vCA1; scale bars = 200 µm. **(B)** Representative microscopic images showing GFP (left), 5-HT (middle) and merge (right) in the CRN; scale bars = 100 µm. **(C-D)** Representative microscopic images showing 5-HT (left), tdTOMATO (middle) and merge (right) in the DRN **(C)** and MRN **(D)** of *Tph2-CreER/Rosa26-LSL-tdTOMATO* mice (with tamoxifen induction at week 8); scale bars = 100 µm. Related to Fig. 3.

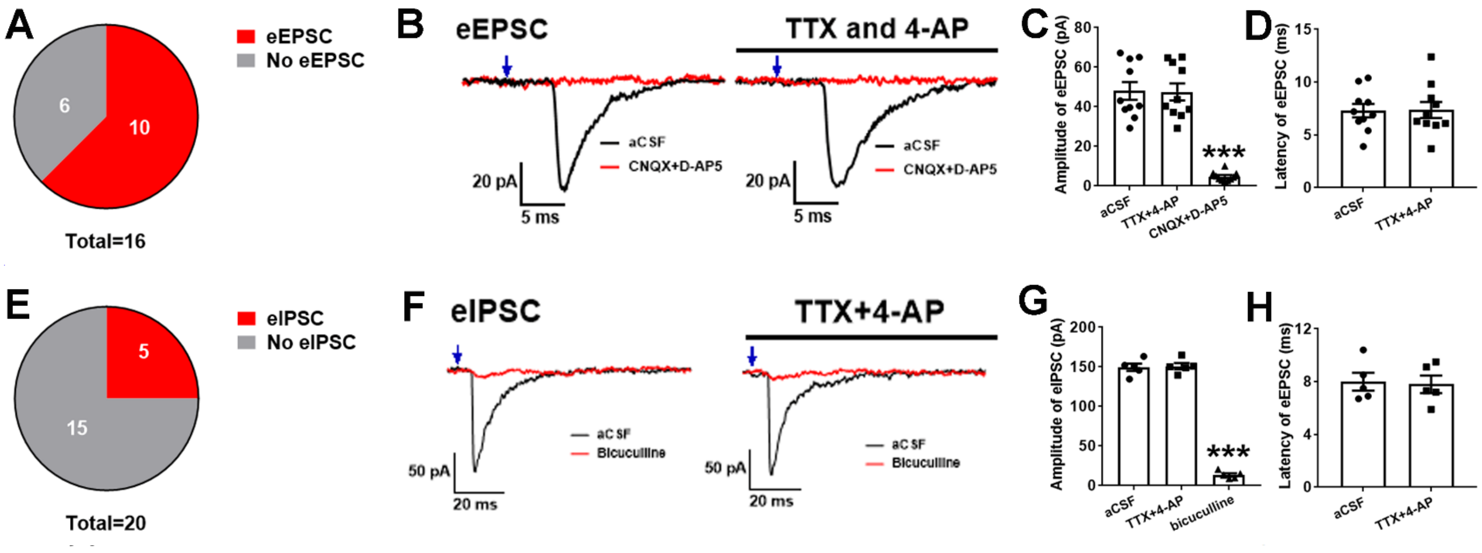

Fig. S6. Midbrain 5-HT neurons project to and activate vCA1 neurons. (A) Percentage of tested vCA1 neurons showing light-evoked EPSCs. **(B)** Representative EPSC traces induced by blue light stimulation (473 nM, 40 mW, 10 ms pulse) in the absence or the presence of various inhibitors. **(C-D)** Amplitude **(C)** and latency **(D)** of light-evoked EPSC. Results are shown as mean ± SEM with individual data points. ***, P<0.001 in one way ANOVA followed by Tukey's test (N= 10 neurons from 3 mice per group). **(E)** Percentage of tested vCA1 neurons showing light-evoked IPSCs. **(F)** Representative IPSC traces induced by blue light stimulation in the absence or the presence of glutamate receptor inhibitors (30 µM CNQX and 30 µM D-AP5) or the $GABA_A$ receptor inhibitor (50 µM bicuculline). **(G-H)** Amplitude **(G)** and latency **(H)** of light-evoked IPSC. Results are shown as mean ± SEM with individual data points. ***, P<0.001 in one way ANOVA followed by Tukey's test (N= 5 neurons from 3 mice per group). Related to Fig. 3.

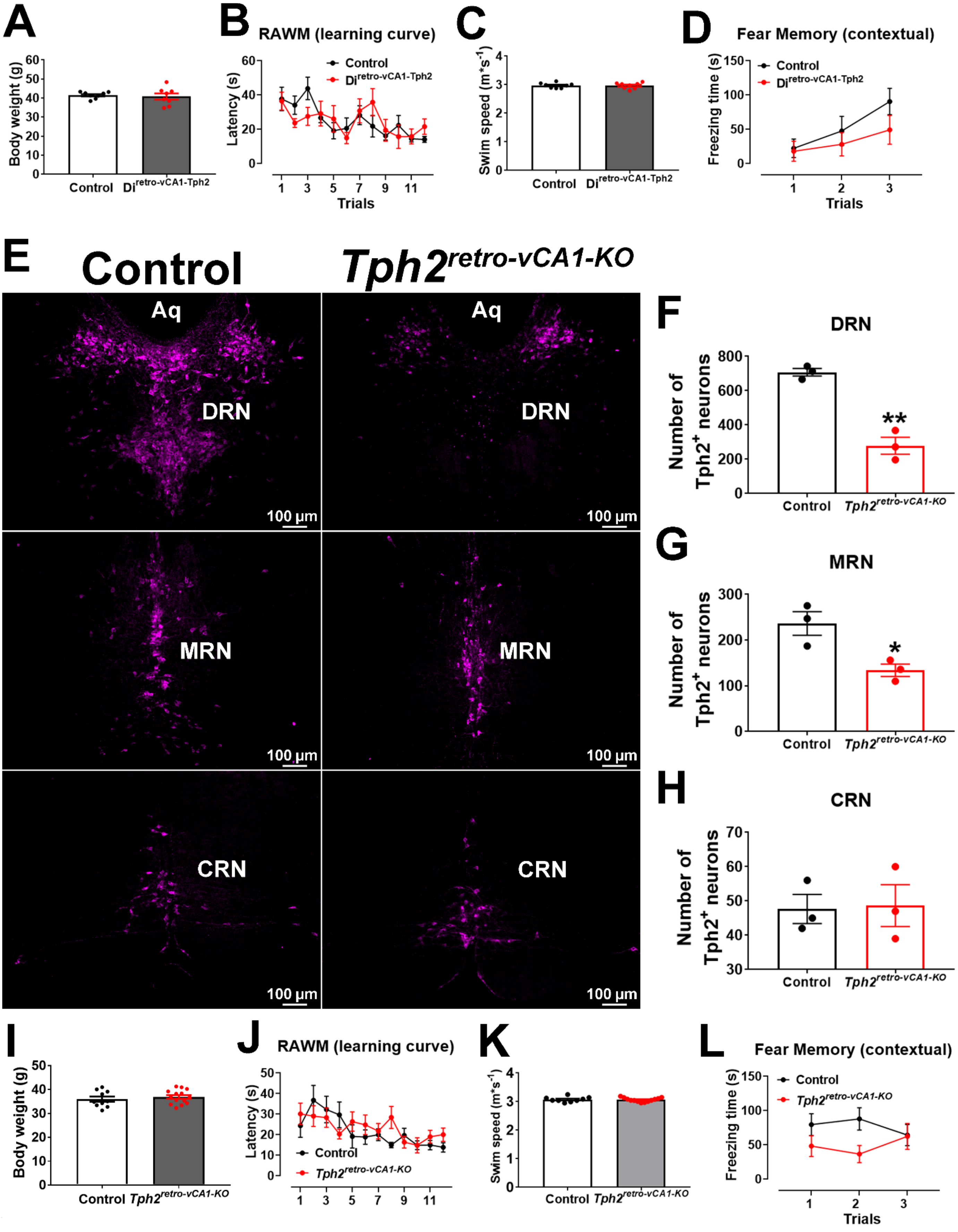
A
Body weight (g)
Control
Di^retro-vCA1-Tph2
B
RAWM (learning curve)
Control
Di^retro-vCA1-Tph2
Latency (s)
Trials
C
Swim speed (m*s^-1)
Control
Di^retro-vCA1-Tph2
D
Fear Memory (contextual)
Control
Di^retro-vCA1-Tph2
Freezing time (s)
Trials
E
Control
Tph2^retro-vCA1-KO
Aq
DRN
MRN
CRN
100 μm
F
DRN
Number of Tph2+ neurons
Control
Tph2^retro-vCA1-KO
G
MRN
H
CRN
I
Body weight (g)
Control
Tph2^retro-vCA1-KO
J
RAWM (learning curve)
Latency (s)
Control
Tph2^retro-vCA1-KO
K
Swim speed (m*s^-1)
Control
Tph2^retro-vCA1-KO
L
Fear Memory (contextual)
Control
Tph2^retro-vCA1-KO
Freezing time (s)
Trials

Fig. S7. 5-HTergic projections to the vCA1 regulate memory. (A) Body weight of male control and *Di*$^{retro\text{-}vCA1\text{-}Tph2}$ mice (4 months) at the time of memory tests. Results shown as mean ± SEM with individual data points (N=8 mice). **(B)** Latency to reach the platform during the 12 RAWM training trials. Results shown as mean ± SEM. **(C)** Swim speed during the RAWM. Results shown as mean ± SEM with individual data points. **(D)** Performance in the fear conditioning test reported as freezing time in response to contextual cues. Results shown as mean ± SEM. **(E)** Representative microscopic images showing Tph2-expressing neurons in the DRN, MRN and CRN of control and *Tph2*$^{retro\text{-}vCA1\text{-}KO}$ mice; scale bar = 100 µm. **(F-H)** Quantification of neurons that were Tph2 positive in the DRN **(F)**, the MRN **(G)** and the CRN **(H).** Results are shown as mean ± SEM with individual points. *, P<0.05 and **, P<0.01 in two-tailed unpaired test (N= 3 mice per group). **(I)** Body weight of male control and *Tph2*$^{retro\text{-}vCA1\text{-}KO}$ mice (6 months) at the time of memory tests. Results shown as mean ± SEM with individual data points (N=10 or 14 mice). **(J)** Latency to reach the platform during the 12 RAWM training trials. Results shown as mean ± SEM. **(K)** Swim speed during the RAWM. Results shown as mean ± SEM with individual data points. **(L)** Performance in the fear conditioning test reported as freezing time in response to contextual cues. Results shown as mean ± SEM. Related to Fig. 4.

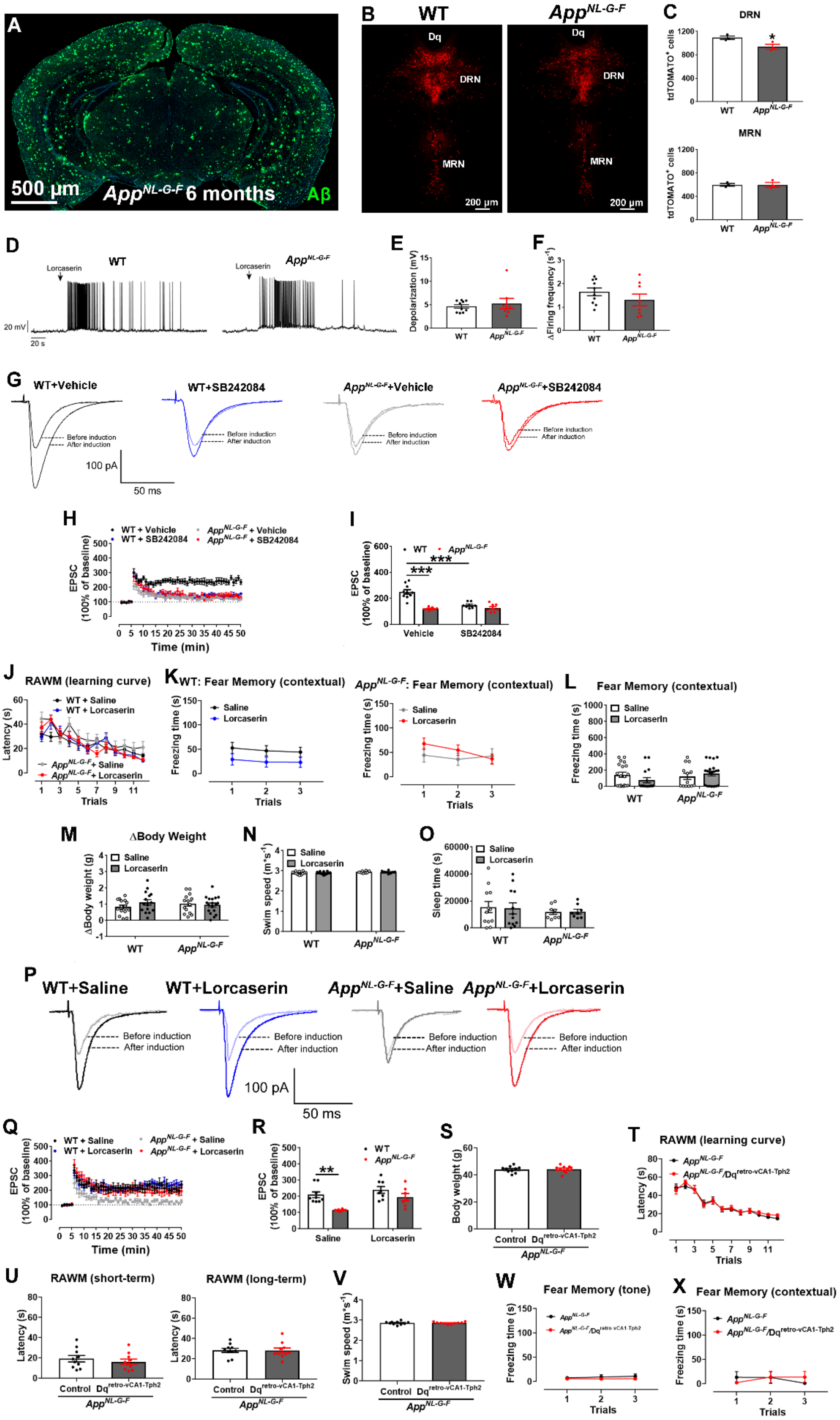

Fig. S8. The 5-HT2cR agonist improves vCA1 neural plasticity and memory in *App$^{NL\text{-}G\text{-}F}$* mice. (A) A representative microscopic image showing Aβ immunoreactivity (green) in a coronal section of a 6-month old male *App$^{NL\text{-}G\text{-}F}$/Tph2-CreER/Rosa26-LSL-tdTOMATO* mouse. **(B)** Representative microscopic images showing 5-HT neuron cell bodies (labelled through

Tph2-CreER/Rosa26-LSL-tdTOMATO allele induced by tamoxifen at 8 weeks) in the DRN and MRN of 6-month old WT or *App*$^{NL\text{-}G\text{-}F}$ mice. **(C)** Quantification of tdTOMATO^{+} neurons in the DRN and MRN. Results are shown as mean ± SEM with individual data points. *, P<0.05 in two-sided unpaired t-test (N=3 mice per group). **(D)** Representative current clamp traces in vCA1 neurons from WT or *App*$^{NL\text{-}G\text{-}F}$ mice treated with lorcaserin (100 µM, 5 seconds puff). **(E-F)** Magnitude of lorcaserin-induced depolarization **(E)** and increase in firing frequency **(F)**. Results are shown as mean ± SEM with individual data points (N=8 or 10 neurons from 3 mice per group). **(G)** Representative EPSC traces before and after LTP induction in vCA1 neurons from WT and *App*$^{NL\text{-}G\text{-}F}$ mice treated with vehicle or SB242084. **(H)** Magnitude of EPSC elevations before and after LTP induction. **(I)** Averaged EPSC elevations during 45-50 min in panel **(H)**. Results shown as mean ± SEM with individual data points. ***, P<0.001 in two-way ANOVA analysis followed by Sidak tests (N= 7, 8 or 10 neurons from 3 mice per group). **(J)** Latency to reach the platform during the 12 RAWM training trials by male saline or lorcaserin-treated WT and *App*$^{NL\text{-}G\text{-}F}$ mice (6 months). **(K)** Performance in the fear conditioning test reported as freezing time in response to contextual cues. Results shown as mean ± SEM. **(L)** Sum of freezing time in **(K)** in each group. Results shown as mean ± SEM with individual data points. **(M)** Changes in body weight of male saline or lorcaserin-treated WT and *App*$^{NL\text{-}G\text{-}F}$ mice (6 months) at the time of memory tests. Results shown as mean ± SEM with individual data points (N=14, 15 or 18 mice). **(N)** Swim speed during the RAWM. Results shown as mean ± SEM with individual data points. **(O)** Sleep time per day. Results shown as mean ± SEM with individual data points. **(P)** Representative EPSC traces before and after LTP induction in vCA1 neurons from WT and *App*$^{NL\text{-}G\text{-}F}$ mice injected with saline or lorcaserin. **(Q)** Magnitude of EPSC elevations before and after LTP induction. **(R)** Averaged EPSC elevations during 45-50 min in panel **(Q)**. Results shown as mean ± SEM (N= 7, 8 or 9 neurons from 3 mice per group). **(S)** Body weight of male *App*$^{NL\text{-}G\text{-}F}$ mice (6 months) with or without Dq expressed in vCA1-projecting 5-HT neurons at the time of memory tests. Results shown as mean ± SEM with individual data points (N=10 mice). **(T)** Latency to reach the platform during the 12 RAWM training trials. Results shown as mean ± SEM. **(U)** Latency to reach the platform in the RAWM test that took place 30 min (short-term) or 24 hr (long-term) after the last training session. Results shown as mean ± SEM with individual data points. **(V)** Swim speed during the RAWM. Results shown as mean ± SEM with individual data points. **(W-X)** Performance in the fear conditioning test reported as freezing time in response to tone **(W)** or contextual **(X)** cues. Results shown as mean ± SEM. Related to Fig. 5.

Table S1. Prospective and Retrospective Memory in young adults carrying loss-of-function HTR2C mutations

	**Memory component tested**	**Self-cued vs. Environmentally-cued**	**Serotonin 2C receptor mutation carriers (age in years, sex)**				
			**F327L (29, F)**	**V2L (28, F)**	**Q282H (29, F)**	**V61I (23, F)**	**A171V (23, F)**
Do you decide to do something in a few minutes time and then forget to do it?	Prospective<br>Short-term	Self-cued	quite often	quite often	quite often	quite often	quite often
Do you fail to recognize a place you have visited before?	Retrospective<br>Long-term	Environmentally-cued	never	never	never	some times	rarely
Do you fail to do something you were supposed to do a few minutes later even though it is there in front of you, like take a pill or turn off the kettle?	Prospective<br>Short-term	Environmentally-cued	quite often	quite often	some times	never	very often
Do you forget something you were told a few minutes before?	Retrospective<br>Short-term	Self-cued	quite often	very often	some times	never	never
Do you forget appointments if you are not prompted by someone else or by a reminder such as a calendar or diary?	Prospective<br>Long-term	Self-cued	quite often	very often	some times	some times	quite often
Do you fail to recognize a character in a radio or television show from scene to scene?	Retrospective<br>Short-term	Environmentally-cued	very often	never	never	quite often	never

Do you forget to buy something you planned to buy, like a birthday card, even when you see the shop?	Prospective<br><br>Long-term	Environmentally-cued	very often	Some times	never	quite often	quite often
Do you fail to recall things that have happened to you in the last few days?	Retrospective<br><br>Long-term	Self-cued	rarely	quite often	very often	quite often	very often
Do you repeat the same story to the same person on different occasions?	Retrospective<br><br>Long-term	Environmentally-cued	quite often	very often	quite often	quite often	very often
Do you intend to take something with you, before leaving a room or going out, but minutes later leave it behind, even though it is there in front of you?	Prospective<br><br>Short-term	Environmentally-cued	quite often	very often	never	some times	very often
Do you mislay something that you have just put down, like a magazine or glasses?	Retrospective<br><br>Short-term	Self-cued	very often	very often	very often	very often	very often
Do you fail to mention or give something to a visitor that you were asked to pass on?	Prospective<br><br>Long-term	Environmentally-cued	Some times	quite often	never	quite often	never
Do you look at something without realizing you have seen it moments before?	Retrospective<br><br>Short-term	Environmentally-cued	quite often	quite often	never	never	very often
If you tried to contact a friend or relative who was out, would you forget to try again later?	Prospective<br><br>Long-term	Self-cued	quite often	quite often	never	quite often	never
Do you forget what you watched on television the previous day?	Retrospective<br><br>Long-term	Self-cued	Some times	quite often	quite often	never	never
Do you forget to tell someone something you	Prospective	Self-cued	quite often	quite often	very often	some times	some times

had meant to mention a few minutes ago?	Short-term						
**Total raw score**			60	62	43	49	52
**T score**			28	26	42	37	34

The Prospective-Retrospective Memory Questionnaire was used to assess effects on short and long-term memory. T scores were calculated according to the methods described by Crawford et al (*21*).

Table S2. Prospective memory test and fluid intelligence scores in unrelated White British participants from UK Biobank 450K exome release

(i) Males	Males, N	Whole cohort 147,346	A7V 1	V61I 14	A171V 139	Q282H 7	F327L 2	T419A 171
Fluid intelligence (FI) score from assessment centre initial visit (Field 20016_i0)	**Males, began FI puzzles, N**	N=48,441	-	N=2	N=51	N=3	N=1	N=57
	Mean FI score	6.30		6.50	6.65	6.00	(<6)[1]	5.77
	p, Mann Whitney U			*p=0.84*	*p=0.16*	*p=0.79*		***p=0.046***
	[3] Linear regression (FI score ~ T419A + age + age^2)							*β_{T419A}[95% CI]= -0.48[-1.04-0.08], p=0.095*
Prospective memory (PM) test from assessment centre initial visit (Field 20018_i0)	**Males with available PM test result, N**	N=49,477	-	N=3	N=52	N=3	N=1	N=58
	Correct recall on first attempt, n (%)	39,772 (80.4%)		3 (100%)	43 (82.7%)	2 (66.7%)	1 (100%)	41 (70.7%)
	Correct recall on second attempt, n (%)	7,493 (15.1%)		-	5 (9.6%)	1 (33.3%)	-	9 (15.5%)
	Instruction not recalled, either skipped or incorrect, n (%)	2,212 (4.5%)		-	4 (7.7%)	-	-	8 (13.8%)
	p, Fisher's exact			*p=1.00*	*p=0.29*	*p=0.48*	*p=1.00*	***p=0.0086***
	[2] Logistic regression (Recalled ~ T419A + age + age^2)							*β_{T419A}= -1.15±0.39, exp(β_{T419A})[95% CI]= 0.32[0.16-0.73],* ***p=0.0028***
Mental Health (MH) online questionnaire, self-reported professional diagnoses (Field 20544): Mental health problems ever diagnosed by a professional; Depression="Depression"; Anxiety="Anxiety, nerves or generalized anxiety disorder".	**Males, completed MH questionnaire, N**	N=46,137	-	N=5	N=51	N=3	-	N=53
	Depression, n (%)	7,243 (15.7%)		2 (40%)	9 (17.6%)	0 (0%)		9 (17.0%)
	OR [95% CI]			3.6 [0.3-31]	1.2 [0.5-2.4]	0		1.1 [0.5-2.3]
	p, Fisher's exact			*0.18*	*0.70*	*1.00*		*0.71*
	Anxiety, n (%)	4,969 (10.8%)		2 (40%)	8 (15.7%)	0 (0%)		8 (15.1%)
	OR [95% CI]			5.5 [0.5-48]	1.5 [0.6-3.3]	0		1.5 [0.6-3.2]
	p, Fisher's exact			*p=0.093*	*p=0.26*	*p=1.00*		*p=0.27*

(ii) Females	Females, N	Whole cohort 171,019	A7V 6	V61I 39	A171V 436	Q282H 10	F327L 10	T419A 355
Fluid intelligence (FI) score from assessment centre initial visit (Field 20016_i0)	**Females, began FI puzzles, N**	N=55,525	N=1	N=14	N=127	N=6	N=2	N=123
	Mean FI score	6.09	(>6)[1]	6.79	5.84	6.83	7.50	5.97
	p, Mann Whitney U			*p=0.32*	*p=0.09*	*p=0.46*	*p=0.31*	*p=0.74*
Prospective memory (PM) test from assessment centre initial visit (Field 20018_i0)	**Females with available PM test result, N**	N=56,593	N=1	N=15	N=129	N=6	N=2	N=124
	Correct recall on first attempt, n (%)	45,154 (79.8%)	1 (100%)	11 (73.3%)	101 (78.3%)	4 (66.7%)	2 (100%)	105 (84.7%)
	Correct recall on second attempt, n (%)	9,587 (16.9%)	-	4 (26.7%)	23 (17.8%)	2 (33.3%)	-	18 (14.5%)
	Instruction not recalled, either skipped or incorrect, n (%)	1,852 (3.3%)	-	-	5 (3.9%)	-	-	1 (0.8%)
	p, Fisher's exact			*p=0.59*	*p=0.77*	*p=0.41*	*p=1.00*	*p=0.23*
Mental Health (MH) online questionnaire, self-reported professional diagnoses (Field 20544): Mental health problems ever diagnosed by a professional; Depression="Depression"; Anxiety="Anxiety, nerves or generalized anxiety disorder".	**Females, completed MH questionnaire, N**	N=58,220	N=5	N=14	N=149	N=2	N=3	N=120
	Depression, n (%)	15,016 (25.8%)	2 (40.0%)	4 (28.6%)	34 (22.8%)	2 (100%)	0 (0%)	32 (26.7%)
	OR [95% CI]		1.9 [0.2-17]	1.2 [0.3-4.0]	0.9 [0.6-1.3]	inf [0.5-inf]	0 [0.0-7.0]	1.0 [0.7-1.6]
	p, Fisher's exact		*p=0.61*	*p=0.77*	*p=0.45*	*p=0.067*	*p=0.57*	*p=0.83*
	Anxiety, n (%)	9,830 (16.9%)	0 (0%)	3 (21.4%)	14 (9.4%)	2 (100%)	0 (0%)	15 (12.5%)
	OR [95% CI]		0	1.3 [0.2-5.1]	0.5 [0.3-0.9]	inf [0.9-inf]	0 [0.0-12]	0.7 [0.4-1.2]
	p, Fisher's exact		*p=0.60*	*p=0.72*	***p=0.012***	***p=0.029***	*p=1.00*	*p=0.22*

Footnotes. P-values are nominal. Nominal p-values < 0.05 are highlighted in **bold** font. Displaying GOOS variants previously investigated for loss-of-function in cells [20] with at least one carrier among UK Biobank unrelated White British exomes for whom prospective memory tests and fluid intelligence scores were available (excludes V2L, N6K, C266R, I97V, V208M, N213T, S260G). The proportion of self-reported professional diagnoses of depression or anxiety were subsequently inspected among carriers of each variant. [1] Summarised to obscure individual-level data. [2,3] The effect of T419A carrier status among males was subsequently tested with covariates age and age^2 for [2] FI score and [3] the odds of correct recall ('Recalled').
